# Effect of SO_2_ and SO_3_ Exposure to Cu-CHA on Surface Nitrate and N_2_O Formation for NH_3_–SCR

**DOI:** 10.1021/acsengineeringau.4c00004

**Published:** 2024-05-31

**Authors:** Joonsoo Han, Joachim D. Bjerregaard, Henrik Grönbeck, Derek Creaser, Louise Olsson

**Affiliations:** † Department of Chemistry and Chemical Engineering, Competence Centre for Catalysis, Chalmers University of Technology, Göteborg 41296, Sweden; ‡ Department of Physics and Competence Centre for Catalysis, Chalmers University of Technology, Göteborg 41296, Sweden

**Keywords:** SO_2_/SO_3_-exposure, Cu-CHA, TPD (temperature-programmed desorption), AN (ammonium nitrate), N_2_O

## Abstract

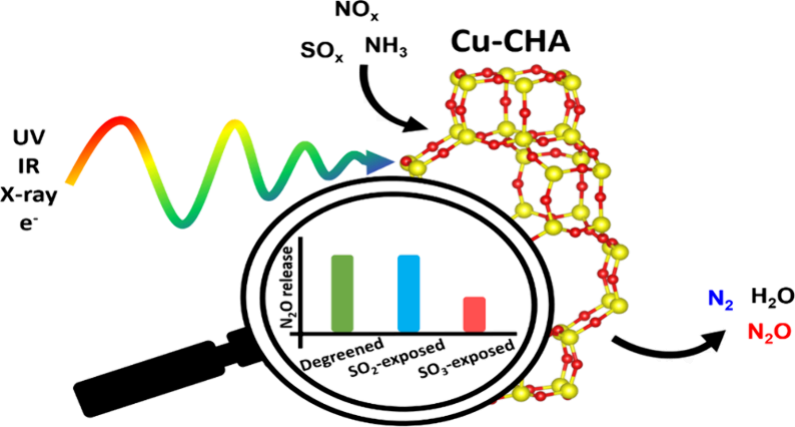

We report effects of SO_2_ and SO_3_ exposure on ammonium nitrate (AN) and N_2_O formation in Cu-CHA used for NH_3_–SCR. First-principles calculations and several characterizations (ICP, BET, XRD, UV–vis–DRS) were applied to characterize the Cu-CHA material and speciation of sulfur species. The first-principles calculations demonstrate that the SO_2_ exposure results in both (bi)sulfite and (bi)sulfate whereas the SO_3_ exposure yields only (bi)sulfate. Furthermore, SOx adsorption on framework-bound dicopper species is shown to be favored with respect to adsorption onto framework-bound monocopper species. Temperature-programmed reduction with H_2_ shows two clear reduction states and larger sulfur uptake for the SO_3_-exposed Cu-CHA compared to the SO_2_-exposed counterpart. Temperature-programmed desorption of formed ammonium nitrate (AN) highlights a significant decrease in nitrate storage due to sulfur species interacting with copper sites in the form of ammonium/copper (bi)bisulfite/sulfate. Especially, highly stable sulfur species from SO_3_ exposure influence the NO_2_–SCR chemistry by decreasing the N_2_O selectivity during NH_3_–SCR whereas an increased N_2_O selectivity was observed for the SO_2_-exposed Cu-CHA sample. This study provides fundamental insights into how SO_2_ and SO_3_ affect the N_2_O formation during ammonium nitrate decomposition in NH_3_–SCR applications, which is a very important topic for practical applications.

## 1 Introduction

NH_3_-assisted selective catalytic reduction (SCR) has been extensively applied for NOx emission control in stationary and mobile applications (e.g., power plants, automobiles, and ships). Copper-exchanged chabazite (Cu-CHA), composed of a small pore entrance (eight-membered rings, 3.8 × 3.8 Å) and double six-membered rings, has been successfully commercialized in exhaust after treatment systems (EATS) for diesel combustion thanks to its high activity and hydrothermal stability at low and high temperatures, respectively.^1−3^ Nitrous oxide (N_2_O) emissions, which may be formed via side reactions during NOx reduction, must be regulated because of their ∼300 times stronger greenhouse gas potential than carbon dioxide (CO_2_) and depletion of the stratospheric ozone.^4,5^ As a result, there is a great deal of interest in understanding the fundamental mechanisms of N_2_O formation during NH_3_–SCR of NOx to improve N_2_ selectivity and minimize N_2_O emissions.

Utilization of biomass in fuels is indispensable to achieve sustainability goals and reduce fossil fuel usage, resulting in lower fossil carbon dioxide (CO_2_) emissions.^6^ However, combustion of petrol, diesel, and biofuels and usage of engine lubricants result in exhaust containing common poisons such as alkali metals, sulfur, phosphorus, and ash, causing contamination of the catalyst and lowering overall deNOx performance in EATS.^7,8^ Among the contamination sources, sulfur has been measured to have severe effects on the activity of the SCR catalysts.^9−16^ Sulfur is present in the form of sulfur oxides (SOx) after combustion of the fuels and originates mainly from the fuel. SO_2_-exposed Cu-CHA shows reduced deNOx performance compared to degreened Cu-CHA.^11,12^ SO_3_-exposed Cu-CHA, however, shows critically reduced deNOx performance and involves larger sulfur storage compared to SO_2_-exposed Cu-CHA.^9,17^ Thus, there is a strong demand to find strategies to reduce the effects of sulfur to maintain the catalytic activity during the lifetime of the catalyst.^7^ In modern diesel EATS, a diesel oxidation catalyst (DOC) plays an important role to increase the NO_2_ content, which promotes NO_X_ reduction and passive regeneration of the SCR and diesel particulate filter (DPF), respectively.^18^ Unfortunately, SO_2_ is mainly oxidized via the DOC and coated DPF, resulting in SO_3_ and H_2_SO_4_ (formed with water via SO_3_+H_2_O → H_2_SO_4_) in the exhaust gas mixture.^18,19^

Although Europe follows the fuel standard (EN590) limiting the sulfur concentration to a maximum of 10 ppm(w), a substantial amount of sulfur (around 2.5 kg of S) could reach the catalysts in the EATS for heavy-duty applications during its lifespan.^8,20^ Periodic regeneration of the DPF (also referred to as active regeneration) can cause temperatures reaching over 650 °C in the filter, which means that also the downstream SCR catalyst is exposed to these high temperatures.^21^ During the high temperature, the accumulated surface sulfur species can leave the surface of the oxidation catalysts (DOC/DPF) whereupon the resulting sulfur oxides (SO_2_, SO_3_, and H_2_SO_4_) reach the SCR catalyst. According to the SOx-TPD with an EATS configuration (SCR+DOC+DPF+SCR+ASC), the DOC is the main contributor of the SO_3_/H_2_SO_4_ generation.^22^ With a sulfated SCR catalyst, the adsorbed sulfur species cause CuO formation during the desulfation event. This desulfation event can cause critical loss of the SCR active copper ions to inactive Cu_*x*_O_*y*_ species without severe zeolite structure deterioration.^23^

NH_3_–SCR reactions undergo different reaction schemes depending on the NO and NO_2_ ratio in the exhaust gas mixture.^24,25^ With only NO present, the NH_3_–SCR reaction includes O_2_ in the reaction stoichiometry and the reaction follows the so-called standard SCR reaction.^24^ The SCR catalyst can be exposed to NO_2_, which can be formed via an oxidation catalyst located upstream of the SCR catalyst. When using equimolar NO/NO_2_ amounts, the NOx reduction rate is enhanced compared to standard SCR, in the so-called fast SCR reaction.^25^ However, when the NO_2_ content is high, the slow NO_2_ SCR reaction occurs, which reduces the reaction rate even when compared to the standard SCR reaction.^25^

Standard SCR: 4NO + 4NH_3_ + O_2_ → 4N_2_ + 6H_2_O

Fast SCR: NO + NO_2_ + 2NH_3_ → 2N_2_ + 3H_2_O

NO_2_ SCR (slow SCR): 3NO_2_ + 4NH_3_ → 3.5N_2_ + 6H_2_O

A variety of Cu^+^/Cu^2+^ active sites have been identified depending on the temperature and gas composition in Cu-CHA. At a low temperature, H_2_O or NH_3_ solvation of the isolated copper sites causes framework-detached mobile Cu species such as Cu^2+^(H_2_O)_*x*_, Cu^2+^(OH)(H_2_O)_*x*_, Cu_2_^2+^O_2_(NH_3_)_4_, and Cu^+^(NH_3_)_2_.^26^ At a high temperature, H_2_O or NH_3_ desorption from these mobile Cu-monomer or Cu-dimer species produces framework-bound Cu-monomer (ZCu, ZCu(OH), Z_2_Cu)^26−29^ and oxygen- or hydrogen-containing dimer (Z_2_Cu_2_O, Z_2_Cu_2_O_2_, Z_2_Cu_2_O_*x*_H_*y*_) species.^27,30−32^ The Z denotes a negatively charged framework, and its subscription represents the number of the site. However, under standard SCR conditions at low temperatures (below 250 °C) in the presence of ammonia, the copper species are mobile and the NH_3_-solvated copper dimers ([Cu_2_(NH_3_)_4_O_2_]^2+^) and monomers ([Cu(NH_3_)_2_]^+^) are considered to be key intermediates in the SCR cycle.^2,24,26,33^

Bimodal N_2_O release is measured with a peak at low (below 300 °C) and high (above 300 °C) temperatures during exposure of Cu-CHA in standard SCR conditions.^34−36^ According to the standard SCR mechanism at low temperatures, recent experimental studies have shown that N_2_O is only formed in the reduction half cycle (RHC) during the catalytic turnover of the standard SCR cycle.^37^ Ab initio calculations describing H_2_NNO decomposition over [Cu_2_(NH_3_)_4_OOH]^2+^ complexes explain not only N_2_O formation during the RHC but also the trend of increasing N_2_O formation as a function of Cu content.^36^ Low NO oxidation rates compared to NH_3_–SCR rates on Cu-CHA have been reported by Paolucci et al.,^28^ suggesting that NO oxidation is irrelevant to the standard SCR at low temperatures. In addition, the N_2_O formation rate is an order of magnitude higher than the NO_2_ formation rate.^37^ A pathway where N_2_O is formed via ammonium nitrate (AN) is, thus, not likely to contribute to the N_2_O formation during low-temperature standard SCR. In contrast to the standard SCR, the extent of N_2_O formation is significantly increased when the NO_2_ fraction is increased in the gas mixture^5^ and the 2+ oxidation state of Cu ions is predominantly observed from in situ XAS over Cu-CHA under fast and NO_2_ SCR conditions,^38,39^ suggesting that different reaction schemes are involved with respect to the fast and NO_2_ SCR conditions. Kubota et al.^40^ and Liu et al.^41^ demonstrated that AN is an intermediate species for N_2_O under fast/NO_2_ SCR conditions. Liu et al.^41^ proposed that the fast SCR follows a dual-site mechanism where derived intermediate species (HONO and H_2_NNO) are decomposed at Brønsted sites in a similar manner as the standard SCR scheme suggested by Chen et al.^42^ Han et al.^5^ proposed that the CHA structure is favorable to form surface nitrates due to promotion of the NO_2_ disproportionation within the CHA cage compared to the MFI and BEA structures, and thereby more AN is observed in the CHA structure, followed by MFI and BEA.

After SO_2_ exposure, only small or negligible amounts of stored sulfur has been observed in H forms of zeolites upon sulfur exposure,^43−45^ indicating that SO_2_ storage mainly requires the presence of Cu ions. Physical and chemical poisoning is reported as the sulfur deactivation mechanism in Cu zeolites. SO_2_ and NH_3_ exposure to Cu-CHA shows decreasing micropore volume with increasing S content, suggesting that pore blocking occurs by ammonia interacting with derived sulfur species.^46^ In comparison, only SO_2_ exposure shows no considerable micropore volume decrease with S content.^46^ Temperature-programmed desorption (TPD) tests with an SO_2_+SCR gas composition gives bimodal SO_2_ peaks at low (∼400 °C) and high (↑600 °C) temperatures where ammonium sulfate and Cu sulfate are associated, respectively.^10,45,47−49^ After regeneration, ammonium sulfate is decomposed and thus it causes a reversible deactivation.^9,47^ However, chemically interacting sulfur species at Cu sites (i.e., ZCuHSO_4_) or Al sites (i.e., Al_2_(SO_4_)_3_) are not fully regenerated and these derived sulfur species are highly stable.^9,50^ Furthermore, a fairly high temperature (over 700 °C) is necessary to fully desulfate these highly stable sulfur species over Cu-CHA.^9,50^ A highly thermal regeneration event can result in a hydrothermal aging (HTA) effect that converts the Cu ions to inactive Cu_X_O_*y*_ and CuAlO_*x*_ species as well as CHA structure degradation.^51^ Therefore, high-temperature regeneration can result in irreversible thermal deactivation.

The current mechanistic understanding of the deactivation of SCR catalysts resulting from sulfur is mainly focused on SO_2_ exposure of Cu-CHA. There is a strong consensus that SO_2_ readily interacts with ZCuOH and that the interaction with Z_2_Cu is weaker.^45,48,52−54^ In situ XAS shows that SO_2_ interaction with Cu ions, especially NH_3_-solvated mobile Cu dimers ([Cu_2_(NH_3_)_4_O_2_]^2+^), is particularly sensitive to SO_2_ as compared to other-state Cu ions such as framework-interacting Cu^+^/Cu^2+^ (ZCu/Z_2_Cu) or NH_3_-solvated Cu monomers ([Cu(NH_3_)_2_]^+^).^55^ Another XAS study shows that SO_3_ exposure does not change the local structure of Cu sites within the zeolite framework (i.e., Cu–O coordination number and the Cu–Cu distance, etc.), indicating that Cu sites remain highly dispersed after sulfation as well as following desulfation.^56^ First-principles calculations performed by Bjerregaard et al.^57^ agree with the experimental observations and suggest that the standard SCR deactivation at low temperatures is due to hindered intercage diffusion by ammonium (bi)sulfate species. Thereby, the pairing of Cu-monomer complexes ([Cu(NH_3_)_2_]^+^) via O_2_ activation is hindered, which is a key intermediate step of the oxidation half cycle (OHC) during standard SCR at low temperatures.^34^ The mechanism proposed by Bjerregaard et al.^57^ rationalizes the experimental findings, where the NO conversion and the N_2_O formation are reduced upon SO_2_ exposure of Cu-CHA during the standard SCR at low temperature.^12,58,59^ Mesilov et al.^12^ reported that NOx conversion and N_2_O selectivity are decreased for the SO_2_-exposed Cu-CHA (Si/Al ≈ 7, 3 wt % Cu) during the standard, fast, and NO_2_ SCR reactions. After regeneration, the NOx conversion and N_2_O selectivity were not fully regenerated for the three overall SCR reactions but the N_2_O selectivity was noticeably increased for the NO_2_ SCR compared to the degreened state. A comparative study between the Cu-CHA aged in EATS (270,000–710,000 miles) and HTA+SOx-treated Cu-CHA suggests that sulfur species assists CuO formation without a clear structural damage during desulfation.^23^ In contrast to the SO_2_ exposure, SO_3_ exposure to Cu-CHA results in larger sulfur uptake and more severe irreversible deactivation.^9^

For industrial applications, understanding the fundamentals of SO_X_ chemistry in Cu-CHA as part of an NH_3_ SCR system is critically important to establish an effective desulfation strategy that maintains catalytic activity throughout the usage lifetime of the SCR catalyst. Limited studies are reported with respect to the SO_3_ exposure compared to the SO_2_ exposure effects on Cu-CHA in terms of its deNOx performance and N_2_O release. There are some studies that have reported the SO_2_ exposure effect on the N_2_O formation.^12,37,58,60^ However, to the best of our knowledge, there is no comparative study of the evaluation of the AN and N_2_O formation for the SO_2_- and SO_3_-exposed Cu-CHA, which is the objective of the current study.

## 2 Experimental and Computational Methods

### 2.1 Catalyst Synthesis and Monolith Sample Preparation

Copper-exchanged SSZ-13 (CHA) was prepared by following the hydrothermal synthesis method to acquire a Si to Al molar ratio of ≈15 and ca. 1 wt % Cu. Specifically, 1 wt % Cu was set to favor the exclusive presence of ion-exchanged Cu ions inside CHA cage and avoid possible formation of CuO particles. The acquired Cu/SSZ-13 powders were washcoated on honeycomb monolith substrates (cordierite, 400 cpsi, 15 mm × 20 mm). The specific synthesis and washcoating procedure are described in the Supporting Information (S1. Catalyst synthesis and monolith preparation). The resulting fresh Cu/SSZ-13 powder and monoliths were degreened under standard SCR conditions (400 ppm of NH_3_/NO + 10% O_2_ + 5% H_2_O + Ar as a balance) at 750 °C for 5 h prior to powder sample characterizations and experiments in a synthetic gas bench reactor.

### 2.2 Synthetic Gas Bench (SGB) Reactor Setup

An SGB reactor was used to perform degreening of fresh catalyst powders, SO_*x*_ treatment, and AN temperature-programmed desorption (AN-TPD). Mass flow controllers (MFCs, Bronkhorst) and a controlled evaporator and mixing (CEM, Bronkhorst) system were equipped to provide the required gases (Ar, O_2_, NH_3_, NO, NO_2_, N_2_O, N_2_, H_2_O, and SO_2_). The gas lines were carefully insulated and heated to 191 °C to avoid water condensation and deposition of solids such as AN. Ar was used as an inert balance for all the experiments, and the total flow was 1.2 NL·min^–1^. The required gas mixture was fed to a horizontal quartz tube (inner diameter: 16 mm). The horizontal quartz tube was wrapped with a heating coil and insulated to heat up and control the reactor temperature. The prepared sample was located inside the quartz tube during the pretreatment and experiments.

### 2.3 SO_2_ and SO_3_ Exposure to Degreened Cu-CHA

The prepared monoliths were pretreated in the SGB reactor with SO_2_ and a SO_2_+SO_3_ mixture, which will be referred to as SO_2_ and SO_3_ exposure treatments. First, the monolith was degreened under standard SCR conditions (400 ppm of NH_3_, 400 ppm of NO, 10% O_2_, and 5% H_2_O) at 750 °C for 5 h, following the SO_2_ or SO_3_ treatment sequence, consisting of the following steps: first step: 30 ppm of SO_2_ + base feed (10% O_2_ + 5% H_2_O + Ar) for 1 h; second step: 400 ppm of NH_3_ + base feed for 2 h; third step: 30 ppm of SO_2_ + base feed for 1 h. The reason for applying the ammonia in a separate step between the sulfur steps and not simultaneously with SOx is that feeding SO_3_ and NH_3_ simultaneously causes large ammonium sulfate deposits in the reactor, which could block the lines, even when heating the lines thoroughly. For the SO_2_+SO_3_ treatment, an external device connected upstream from the SGB reactor was used to generate SO_3_. Pt/Al_2_O_3_ (7.5 wt % Pt) was used as an oxidation catalyst in the SO_3_ generator. The specific procedures are described in the Supporting Information (S2. SO_3_ calibration and SO_*x*_-exposure of degreened monoliths). This provided the capability to introduce 24 ppm of SO_3_ + 6 ppm of SO_2_ with base feed to the monolith instead of 30 ppm of SO_2_, as illustrated in Figure S2. Meanwhile, the temperature of the monolith was maintained at 400 °C during the SOx treatment to mimic the SOx exposure environment to the SCR catalyst during periodic regeneration of the DPF. Note that for simplification from here on, the SO_2_ and SO_2_+SO_3_ treatments shall be referred to as SO_2_ and SO_3_ exposure, respectively.

### 2.4 Catalyst Characterization

Elemental analysis was performed with powder forms of the degreened and SO_2_- and SO_3_-exposed samples. The SOx-exposed samples’ washcoats were carefully scraped from the SO_2_- and SO_3_-exposed monoliths. Afterward, the SOx-exposed washcoat samples were used for the characterizations. However, for diffuse reflectance infrared Fourier transform spectroscopy (ICP) and diffuse reflectance infrared Fourier transform spectroscopy (DRIFTS) results of the sulfated samples, crushed monoliths were used.

#### 2.4.1 Catalyst Powder Sample Characterization

Elemental compositions of the fresh catalyst powders were analyzed using inductively coupled plasma sector field mass spectrometry (ICP-SFMS) at ALS Scandinavia AB. For sulfated samples, washcoated sulfated monoliths were crushed well into a fine powder form to acquire the S/Cu molar ratio; afterward, the sulfur content of the sulfated samples was determined.

Powder X-ray diffraction (XRD) was performed with degreened and SO_2_- and SO_3_-exposed samples to confirm the crystalline structure of CHA. A Siemens D5000 diffractometer operating at 40 kV, using the Kα_1_ radiation of a Cu anode as X-ray source (λ = 1.54060 Å), was used for measuring the X-ray diffractogram from the powder samples.

N_2_ physisorption was performed to measure specific surface area using the Brunauer–Emmett–Teller (BET) method of the degreed catalyst powder. A Micromeritics TriStar 3000 instrument was used to measure the N_2_ adsorption/desorption isotherms at 77 K. Prior to the measurements, the H and Cu forms of the degreened catalyst powders were degassed at 225 °C for 8 h.

UV–visible diffuse reflectance spectroscopy (UV–vis-DRS) was performed to investigate the formation of copper and ammonium sulfite/sulfate species. A Lambda 365 (PerkinElmer) double-beam UV visible spectrophotometer was used to acquire the UV spectrum. The system is composed of two different lamps (D2 and tungsten lamp) for measurement within the UV and visible-light regions. The lamp change occurred at 380 nm during the measurement. The light is split into two beams before it interacts with the sample. One of the beams is used as a reference, and the other beam reaches the sample. Two detectors (photodiodes) measured the light intensity, which came from the reference and sample beams simultaneously. Detector and reflectance sphere (sphere diameter: 60 mm, sample port aperture: 12.5 mm, sphere coating: Barium sulfate) modules were installed. Thereafter, a light trapper and a white standard were used for the calibration of UV intensity. Finally, the prepared catalyst powder was transferred to a powder cell for measuring the powder sample. The sample was scanned from 1100 to 210 at 5 nm slit width and 480 nm·min^–1^ scanning speed.

#### 2.4.2 H_2_ Temperature-Programmed Reduction (H_2_-TPR)

H_2_-TPR was carried out with a differential scanning calorimeter (Sensys DSC calorimeter from Setaram) to investigate the reduction properties of the degreened and SO_2_- and SO_3_-exposed catalyst powders. The powders were carefully sieved to 180–250 μm fraction. Degreened (ca. 50 mg) or SOx-exposed (ca. 26 mg) samples were loaded into a quartz tube located inside the DSC, and 0.2% H_2_/Ar was continuously fed at 20 N mL·min^–1^ during the experiment. Initially, the temperature was set at 25 °C for 20 min and then was ramped up to 800 °C at 10 °C·min^–1^; thereafter, 800 °C was maintained for 20 min. Note that there was no pretreatment done prior to H_2_-TPR. In the meantime, the outlet gases from the DSC were monitored with mass spectrometry (Hidden HPR-20 QUI MS) for H_2_ (*m*/*e* = 2), NH_3_ (15), H_2_O (18), Ar (20), O_2_ (32), H_2_S (34), SO_2_ (64), SO_3_ (80), and H_2_SO_4_ (98).

#### 2.4.3 Ammonium Nitrate Temperature-Programmed Desorption (AN-TPD)

AN-TPD was performed to investigate the effect of SO_2_ and SO_3_ on AN and N_2_O formation in the SGB reactor. 200 ppm of NH_3_/NO_2_ + 5% H_2_O + Ar was fed to the monolith for 90 min at 150 °C, followed by purging with 5% H_2_O+Ar for 90 min. Thereafter, the temperature was increased to 550 °C at 10 °C·min^–1^ and then maintained at 550 °C for 20 min while the monolith was exposed to 5% H_2_O+Ar. Note that no pretreatment was done prior to AN-TPD for the SO_2_- and SO_3_-exposed samples, to avoid the removal of sulfur species. However, the degreed sample was pretreated before AN-TPD. An empty-tube test was done prior to AN-TPD to check for possible AN formation. The test result showed no N_2_O formation during temperature ramping, indicating that the system was sufficiently insulated to prevent any significant AN formation during the NH_3_+NO_2_ feed to the SGB. Meanwhile, the N_2_ (*m*/*e* = 28) signal evolved while 200 ppm of NO_2_/NH_3_ was fed, suggesting that NO_2_–SCR occurred during ionization within MS.

#### 2.4.4 In Situ DRIFTS Measurements

In situ DRIFTS was performed to monitor surface nitrite/nitrate and NH_3_ interaction with the prepared samples (i.e., degreened and SO_2_- and SO_3_-exposed samples). A VERTEX 70 FTIR spectrometer (Bruker) equipped with a liquid N_2_-cooled mercury cadmium telluride (MCT) detector was used to acquire the IR spectra. Degreened powder samples or crushed SOx-exposed monoliths were loaded into a sample cup inside a reaction cell. A KBr bed was set on the bottom of the sample cup, and then the remaining volume of the sample cup was filled with sample. The reaction cell was covered well and mounted with a Ca_2_F window. A thermocouple was installed at the center of a sample bed to monitor the sample bed temperature during the measurement. The temperature of the sample bed was controlled at 150 °C with a PID regulator (Eurotherm 2416), and MFCs (Bronkhorst Hi-Tech) were set to supply the required gas mixture to the reaction cell. 1% H_2_O + Ar was fed as base feed throughout the measurement. The required gases were fed in the following steps: (1) heating to 150 °C in base feed only and (2) 400 ppm of NO_2_ + base feed for 1.5 h. Meanwhile, DRIFTS spectra were measured (accumulation of 60 scans, resolution of 4 cm^–1^) over time under 100 N mL·min^–1^ of total flow. The outlet gas leaving the reaction cell was continuously monitored with mass spectrometry (Hidden HPR-20 QUI MS). Note that the reported temperature in the DRIFTS test was monitored by a thermocouple located at the center of the sample bed. However, the heating plate located under the sample cup was set at 242 °C. Thus, a temperature gradient resulting from thermal radiation was present and expected.

#### 2.4.5 First-Principles Calculations

Spin-polarized density functional theory (DFT) calculations were performed using the Vienna Ab initio Simulation Package (VASP).^61,62^ The Kohn–Sham orbitals were expanded with plane waves with a 480 eV cutoff value, and the interaction between the core and valence electrons was described by the projector augmented wave (PAW)^63,64^ method. The valence electrons treated for each atom were Cu(11), Si(4), S(6), Al(3), O(6), and H(1). The k-point sampling was restricted to the gamma point. The Perdew–Burke–Ernzerhof (PBE)^65^ functional was used to describe the exchange-correlation effects. To account for van der Waals interactions, the Grimme 3D approach^66^ was applied and a Hubbard *U*-term was used to describe the localized Cu 3d electrons, with the *U* value set to 6 eV.

The convergence criteria for the self-consistent field (SCF) loop was set to 10^–5^ eV, and structures were considered to be at a minimum if the norm of all forces was less than 0.02 eV/Å. The transition states were located using the Climbing Image Nudged Elastic Band (CI-NEB) approach^67,68^ and was confirmed by a single imaginary frequency. Vibrational analysis was performed using the finite difference method. To sample low energy configurations, ab initio molecular dynamics (AIMD) simulations were carried out at 300 K using a Nosé–Hoover thermostat in the NVT ensemble.^69,70^ Several structures along the trajectories were extracted and relaxed to determine the low-energy structures. Bader charge analysis was performed using the implementation from the Henkelman group.^71,72^ To describe the CHA cage, a hexagonal unit cell containing 36 Si and 72 O was used with the experimental lattice constants (13.8026 Å, 13.8026 Å, 15.0753 Å). For each Cu ion introduced into the unit cell, one Si atom was replaced by one Al atom for charge compensation.

## 3 Results

### 3.1 First-Principles Calculation on SO_*x*_ Adsorption over Cu Sites

Two experimentally observed resting states of the Cu^2+^ ions in CHA are Z_2_Cu and ZCuOH.^26,73^ Depending on the sample composition and pretreatment, other Cu^2+^ sites may form and it is important to consider which Cu sites may be present under the conditions investigated. In our study, the samples have been exposed to SO_*x*_ at 400 °C in the presence of O_2_ and H_2_O. At 400 °C, Cu^2+^ is mainly bound to the framework and we would therefore expect to have both Z_2_Cu and ZCuOH present. In addition, the presence of O_2_ could lead to the formation of Cu dimers as copper ions can possibly migrate, forming a pair of Cu^2+^ ions, which can adsorb O_2_ forming Z_2_CuO_2_Cu complexes.^74^ Other similar dimer sites have been proposed such as Z_2_Cu–O–Cu and Z_2_CuO_2_H_2_Cu.^30,75,76^ The Cu sites that will be investigated in the computational study are Z_2_Cu, ZCuOH, and Z_2_CuO_2_Cu.

To explore the effect of SO_2_ and SO_3_, we calculated reaction pathways for the formation of CuHSO_3_ and CuHSO_4_ on Z_2_Cu, ZCuOH, and Z_2_CuO_2_Cu. The reaction of SO_2_ on Z_2_Cu and ZCuOH has previously been studied using DFT calculations by Jangjou et al.,^45^ and they found that the addition of SO_2_ results in the formation of more stable CuHSO_3_ complexes on ZCuOH than on Z_2_Cu. In addition, they found that the formation of CuHSO_3_ exhibited a lower activation barrier when it was formed on ZCuOH.^45^ Furthermore, the stability of CuHSOx complexes from the reaction of SO_2_ and SO_3_ with ZCuOH has been considered with DFT calculations^13^ and it was found that adsorption of SO_3_ gives rise to more stable complexes than SO_2_ adsorption. However, the understanding of the reaction of SO_3_ with ZCuOH and Z_2_Cu and the role of Z_2_CuO_2_Cu upon sulfur exposure is currently limited. Herein, we investigate the reaction pathway for the combinations of SO_2_ and SO_3_ on Z_2_Cu, ZCuOH, and Z_2_CuO_2_Cu, which are Cu sites expected to be present during the first sulfation step in our study.

**Figure 1:**
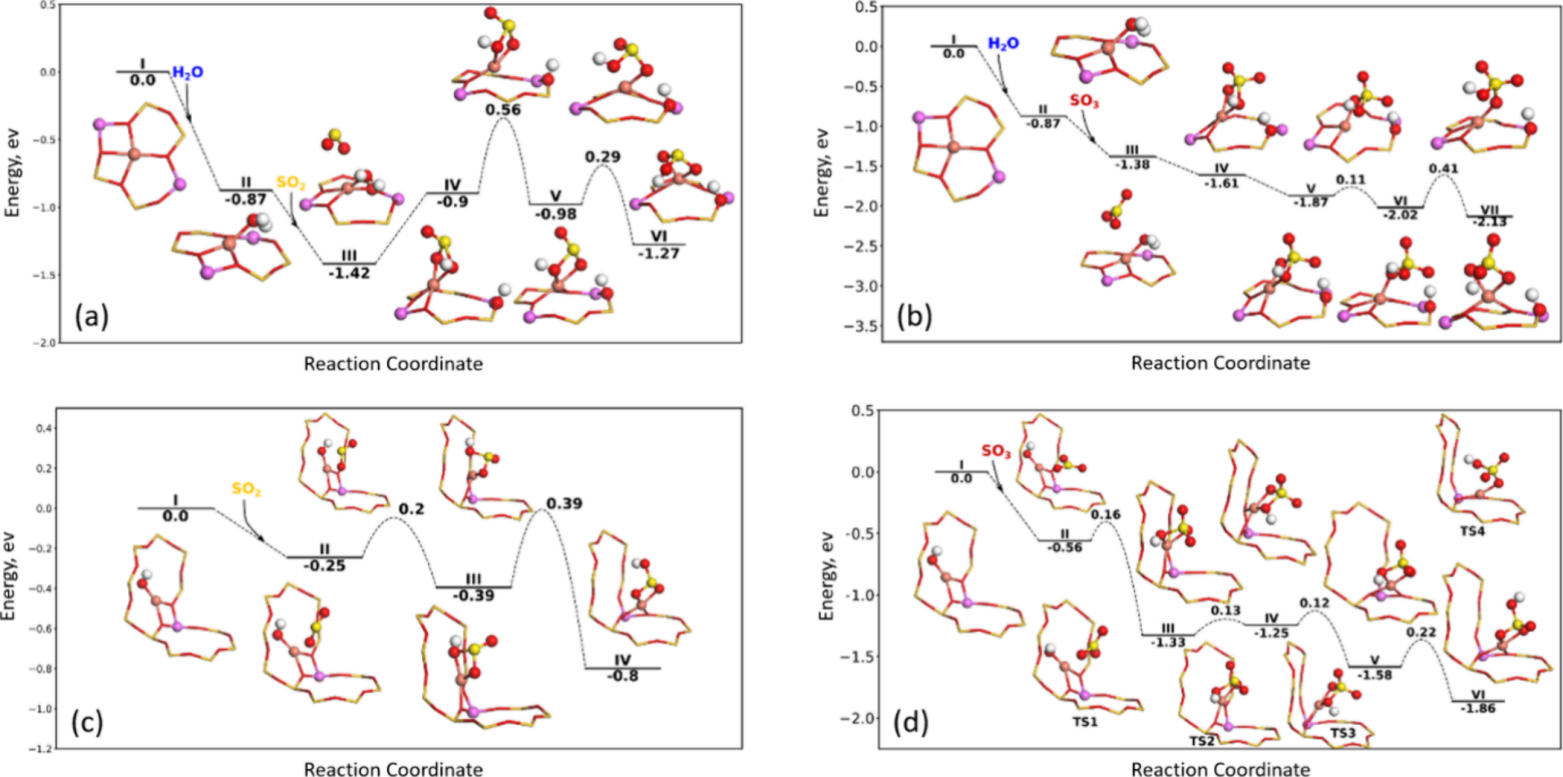
Energy landscapes for SO_2_ and SO_3_ adsorption onto Z_2_Cu (a, b) and ZCuOH (c, d) and the subsequent formation of CuHSO_*x*_ complexes. Atomic color codes: H (white), N (blue), O (red), Al (pink), Si (dark yellow sticks), S (yellow), and Cu (bronze).

The reaction of SO_2_ and SO_3_ with Z_2_Cu is calculated in the presence of a single adsorbed H_2_O molecule. If there is no adsorbed H_2_O molecule on Z_2_Cu, it was not possible to form any stable structures with SO_2_ and SO_3_, which is in agreement with a previous observation by Jangjou et al.^45^ for SO_2_ reacting with Z_2_Cu. For the reaction of SO_2_ with Z_2_Cu (Figure 1a), H_2_O adsorbs on the Cu ion with a Cu–O bond length of 1.95 Å and an adsorption energy of 0.87 eV (I → II). The introduction of SO_2_ in the zeolite cage is preferred by 0.55 eV, compared to SO_2_ in the gas phase; however, we do not find any bond to the Cu ion (II → III). Instead, the oxygen in SO_2_ coordinates to Cu with a bond length of 2.49 Å. The first step in the reaction of SO_2_ with Z_2_Cu–H_2_O results in the cleavage of the H–OH bond, forming a CuHSO_3_ complex and a Brønsted acid site (BAS), which is endothermic by 0.52 eV (III → IV). From a Bader charge analysis, sulfur in CuHSO_3_ is in a +4 oxidation state, as is the case for the gas-phase SO_2_. In IV, sulfur binds through an OH group, which is not the preferred configuration. In structure IV, the −OH group in CuHSO_3_ coordinates with the BAS with an SO-HBAS bond length of 3.73 Å. In the next step (IV → V), the CuHSO_3_ complex is rotated. The barrier for the rotation (0.56 eV) is related to the change in which group is coordinating with the Brønsted site. In structure V, an oxygen from CuHSO_3_ is coordinating with the Brønsted site resulting in a slight stabilization. The last step is the breakage of the SOH–Cu bond and formation of a SO–Cu bond (V → VI), which is 0.29 eV lower in energy. The final structure is 0.15 eV higher in energy compared to the case with SO_2_ physisorbed in the cage (structure III). Moreover, as the formation of structure Cu is associated with a high activation barrier, we conclude that the reaction of SO_2_ with Z_2_Cu is not favorable.

SO_3_ can also react with Z_2_Cu, as shown in Figure 1b. The first step is also, in this case, the adsorption of H_2_O (I → II). Introducing SO_3_ into the cage does not result in adsorption; instead, SO_3_ is physisorbed (exothermic by 0.51 eV) with one oxygen coordinated to Cu with a bond length of 3.3 Å (II→ III). SO_3_ can react with H_2_O, splitting the O–H bond in H_2_O and forming a CuHSO_4_ complex and a Brønsted site (III → IV). From Bader charge analysis, sulfur remains in a +6 oxidation state, as is the case for the gas-phase SO_3_. In structure IV, the OH group in CuHSO_4_ is coordinating with HBAS. The next step (IV → V) is related to a change of coordination, resulting in the oxygen of CuHSO_4_ coordinating to HBAS with a bond length of 1.64 Å. The scission of the SOH–Cu bond has a small barrier of 0.11 eV and is preferred by 0.15 eV (V → VI). The last step is the rotation of CuHSO_4_, resulting in the formation of a second SO–Cu bond (VI → VII). This step has a barrier of 0.41 eV and is exothermic by 0.11 eV. The final structure (VIII) is 0.75 eV more stable compared to the case of having physisorbed SO_3_ (structure III). When comparing the reaction of SO_2_ and SO_3_ with Z_2_Cu, it becomes clear that SO_3_ forms ZCuHSO_4_ complexes that are more stable (0.86 eV in difference) than ZCuHSO_3_ complexes formed by the reaction with SO_2_. The highest activation barrier for formation of ZCuHSO_4_ is 0.41 eV (VI → VII), while the effective barrier is 1.46 eV (III → V) for formation of ZCuHSO_3_. This suggests that SO_3_ may result in more severe deactivation than SO_2_. The higher stability of sulfur species in Cu-CHA with the oxidation state of +6 is in agreement with previous DFT studies.^77^

The reaction of SO_*x*_ with ZCuOH can proceed in the absence of H_2_O. The introduction of SO_2_ into the CHA cage is preferred by 0.25 eV (I → II, Figure 1c). Next, SO_2_ can react with ZCuOH forming a CuHSO_3_ complex that is preferred by 0.14 eV and associated with a small barrier of 0.2 eV (II → III). The last step is the rotation of the sulfur complex, resulting in the breakage of a SOH–Cu bond and, at the same time, the formation of a SO–Cu bond. This step has a barrier of 0.39 eV and is 0.41 eV lower in energy. The stable CuHSO_3_ complex (structure IV) is 0.55 eV more stable compared to physisorbed SO_2_ (structure II).

The reaction of SO_3_ with ZCuOH is shown in Figure 1d. Physisorption of SO_3_ is exothermic by 0.56 eV (I → II). SO_3_ can react with ZCuOH forming a CuHSO_4_ complex that is 0.77 eV more stable and associated with a barrier of 0.16 eV (II → III). The following steps are connected to the reorientation of the CuHSO_4_ complex away from the eight-membered ring. The first step has a barrier of 0.13 eV and is endothermic by 0.08 eV (III → IV). The second step has a barrier of 0.12 eV and is exothermic by 0.33 eV (IV → V). The last step is the rotation of the CuHSO_4_ complex, resulting in the breakage of a SOH–Cu bond and the formation of a SO–Cu bond (V → VI). The final CuHSO_4_ complex (structure VI) is 1.3 eV more stable than physisorbed SO_3_ (structure II).

For ZCuOH, the same trend as for Z_2_Cu is observed, where SO_3_ forms more stable sulfur complexes (−1.3 versus −0.55 eV), which is associated with lower activation barriers compared to the SO_2_-derived sulfur complexes (0.22 versus 0.39 eV).

**Figure 2:**
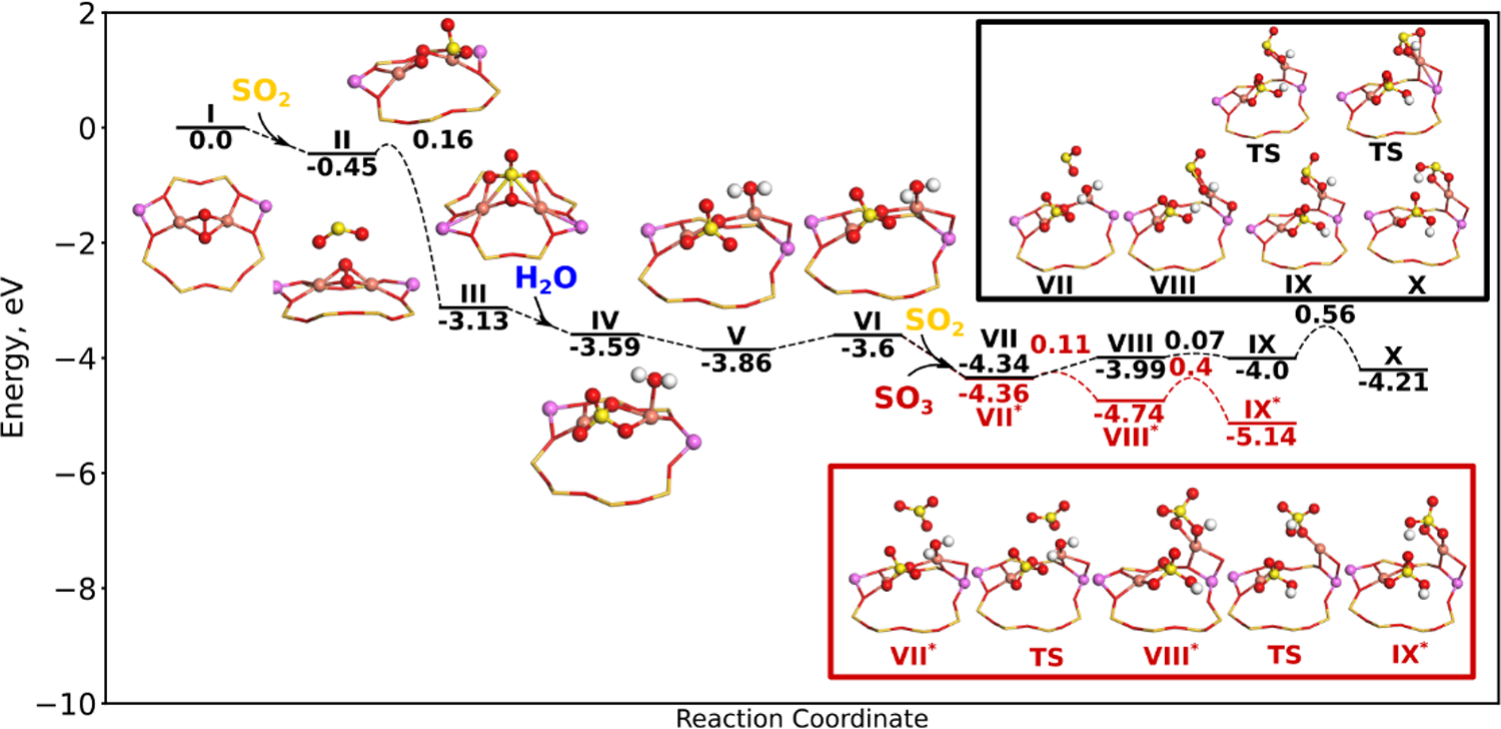
Energy landscape for the reaction of SO_3_ and SO_2_ with Z_2_CuO_2_Cu and the subsequent formation of ZCuHSO_*x*_ complexes. Atomic color codes are as in Figure 1.

As the SOx exposure is performed in the presence of O_2_ at 400 °C, a pair of Cu ions may adsorb O_2_ forming framework-bound peroxo species, Z_2_CuO_2_Cu.^74^ The reaction landscape for the reaction of SO_2_ and SO_3_ with Z_2_CuO_2_Cu is shown in Figure 2. The starting structure I could originate from a pair of Cu^+^ ions that adsorb O_2_.^74^ SO_2_ cannot adsorb directly onto the complex (structure II). Instead, SO_2_ can react (low barrier of 0.16 eV) with the adsorbed O_2_ molecule forming a stable Z_2_Cu(SO_4_)Cu complex (II → III), which is 2.68 eV more stable. During the reaction, sulfur is oxidized from +4 to +6. The strongly exothermic nature of sulfur oxidizing for Cu-CHA has been observed previously for SO_2_ oxidation over the mobile peroxo complex [Cu_2_O_2_(NH_3_)_4_]^2+^.^57^ The Cu ions remain in a +2 oxidation state, supplying the two electrons needed to form the sulfate species (SO_4_^2–^). This complex cannot react directly with SO_2_ or SO_3_; however, in the presence of H_2_O, it is possible to form CuHSO_3_ and CuHSO_4_ complexes. H_2_O can adsorb onto Cu with a binding energy of 0.46 eV (III → IV). The next step is the breakage of a SO–Cu bond (IV → V). Furthermore, a bond between Cu and an oxygen in the framework (O_fw_) is broken and a new Cu–O_fw_ bond is formed. The configuration of structure V is favored for the formation of sulfur complexes. The next step (V → VI) is endothermic by 0.26 eV and is the result of the coordination of hydrogen from H_2_O, which changes from one oxygen to another that is bonded to sulfur. The next steps follow two different paths, which involves the reaction with either SO_2_ or SO_3_ indicated in black or red with a star (*), respectively. SO_2_ (black) can react with H_2_O, splitting the O–H bond of H_2_O and forming a CuHSO_3_ and CuHSO_4_ complex (VII → VIII), which is endothermic with 0.35 eV. The next step (VIII → IX) is the change of the coordination of an OH group bound to sulfur, which is turned away. The last step is the rotation of CuHSO_3_ that leads to the breakage of a SOH–Cu bond and formation of a SO–Cu bond (IX → X). This step has a barrier of 0.56 eV and is exothermic by 0.21 eV. From structure VI, it is also possible to follow the structure denoted with an *, which is the reaction with SO_3_. Here, SO_3_ can react with H_2_O forming two CuHSO_4_ complexes (VII* → VIII*). The formation of CuHSO_4_ is associated with a small barrier of 0.11 eV and is exothermic by 0.38 eV. As in the previous case, the next step involves the rotation of HSO_4_, so the −SOH group is no longer bonded to Cu (VIII* → IX*). The rotation has a barrier of 0.4 eV and is exothermic by 0.4 eV.

From the reaction landscapes, we conclude that more stable sulfur complexes are formed when reacting Z_2_CuO_2_Cu with SO_3_ compared to SO_2_. This is similar to the reactions on Z_2_Cu and ZCuOH. In addition, the activation barriers associated with the reaction with SO_3_ are lower than for SO_2_. Compared to Z_2_Cu and ZCuOH, the first reaction with SO_2_ (II → III) forms more stable complexes suggesting that Z_2_CuO_2_Cu causes more severe deactivation. To form Z_2_CuO_2_Cu, the catalyst must be exposed to O_2_. When SO_2_ is cofed with O_2_, severe deactivation has been experimentally observed,^49^ which is consistent with our calculations. Furthermore, it is possible to oxidize SO_2_ to SO_4_^2–^ over the Z_2_CuO_2_Cu and the presence of sulfur in +6 oxidation states has been observed using X-ray absorption spectroscopy.^78^ The sulfur-derived complexes formed when reacting SOx with Z_2_CuO_2_Cu are the same as those for the cases with Z_2_Cu and ZCuOH. This indicates that even though more sulfur complexes may form on Z_2_CuO_2_Cu, it is difficult to unravel the origin of the species. However, the proximity of two CuHSO_4_ ions could potentially stabilize the complexes. The observation that SO_3_ forms more stable sulfur complexes that are associated with lower barriers compared to SO_2_ for all Cu sites is consistent with the observation of more SOx release in TPD experiments.^9^ In addition, it is observed that SO_3_ in general causes more deactivation of the low-temperature NH_3_–SCR.^9^

### 3.2 Elemental Analysis, N_2_ Physisorption, and XRD Analysis of Powder Cu/SSZ-13

Elemental analysis and N_2_ physisorption were conducted for the prepared copper-exchanged SSZ-13 powders, and the results for the degreened samples are shown in Table S1. SAR (Si/Al molar ratio) was around 14, and the Cu content was 0.83 wt %. The BET surface area decreased (from 711 to 651 m^2^·g^–1^) after impregnation of Cu ions. With respect to sulfated samples, 33 and 159 μmol·g_washcoat_^–1^ of sulfur content were obtained for SO_2_- and SO_3_-exposed samples, respectively. In Figure S3, a comparison of X-ray diffractograms for the degreened H and Cu forms of SSZ-13 shows that the CHA framework structure was well maintained without noticeable diffraction peaks of Cu_*x*_O_*y*_ crystalline particles (36.30 and 38.72° 2θ)^5^ or Cu(OH)_2_ precipitates (12.83 and 25.84° 2θ)^37^ in the sample powders. Note that the fresh sample was degreened in standard SCR conditions at 750 °C for 5 h and a low Cu loading amount was set (ca. 1 wt % Cu) to avoid possible formation of copper-containing particles. This suggests that no or minor amounts of Cu_*x*_O_*y*_ particles were formed and if any was formed; they were below the detection limit of the XRD. It is, thus, assumed that most copper is present as isolated Cu ions interacting with 1 or 2 Al_f_ over the CHA structure of the degreened powder.

**Figure 3:**
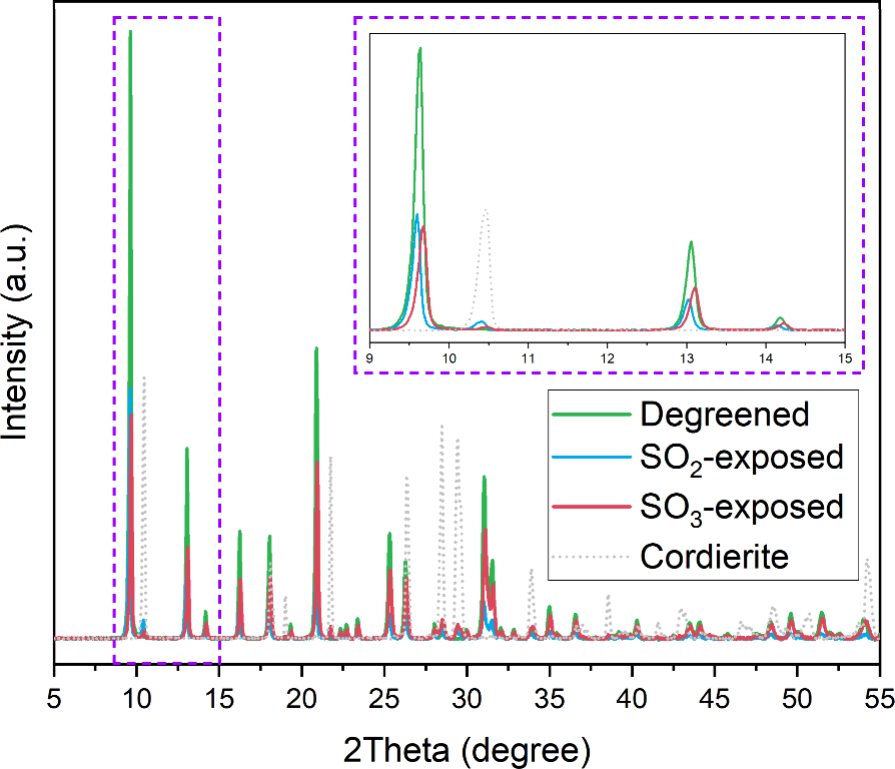
X-ray diffractogram for degreened and SO_2_- and SO_3_-exposed samples. The SO_2_- and SO_3_- exposed samples were scraped off the washcoat from monoliths. In addition, XRD from pure cordierite is shown. The inset illustrates X-ray diffraction peak shifting in the position of crystallographic planes with indices of [1 0 0] (ca. 9.5°), [−1 1 0] (ca. 13°), and [1 1 0] (ca. 14°).

The effect of SO_2_ and SO_3_ exposure to Cu-CHA was examined using SEM and XRD with the following sulfation sequence: (i) SO_2_/SO_3_+O_2_+H_2_O; (ii) NH_3_ + O_2_+H_2_O and (iii) SO_2_/SO_3_+O_2_+H_2_O. We confirmed that no clear changes in crystal morphology from SO_2_- and SO_3_-exposed samples using SEM (see Figure S4) were observed compared to the degreened sample. XRD data are shown in Figure 3, and for the degreened sample, catalyst powder was used while for the SO_2_ and SO_3_-exposed samples, washcoat was scraped off from the monoliths. In addition, XRD of the cordierite is shown and it is clear that the cordierite present in the samples that were scraped off the monoliths can be neglected. The XRD results show shifted diffraction peaks (Figure 3). The inset (dashed purple) in Figure 3 represents the region of crystallographic planes containing [1 0 0] (ca. 9.5°), [−1 1 0] (ca. 13°), and [1 1 0] (14°).^46^ Both SO_2_- and SO_3_-exposed samples caused peak shifting toward either lower or higher angles compared to the degreened sample, respectively. This suggests that SO_2_ and SO_3_ exposure at 400 °C results in changes of the CHA unit cell size but no damaged framework structure according to Bragg’s law.^79^ Decreased peak intensity for SO_2_- and SO_3_-exposed samples indicates that sulfur species were successfully introduced into the CHA unit cell. However, questions remain as to why SO_2_ and SO_3_ caused different trends in the shift of the peak toward lower and higher angles, respectively. This different peak shifting trend implies that different lattice distances resulted from SO_2_ and SO_3_ exposure with NH_3_ oxidation as the middle step.

**Figure 4:**
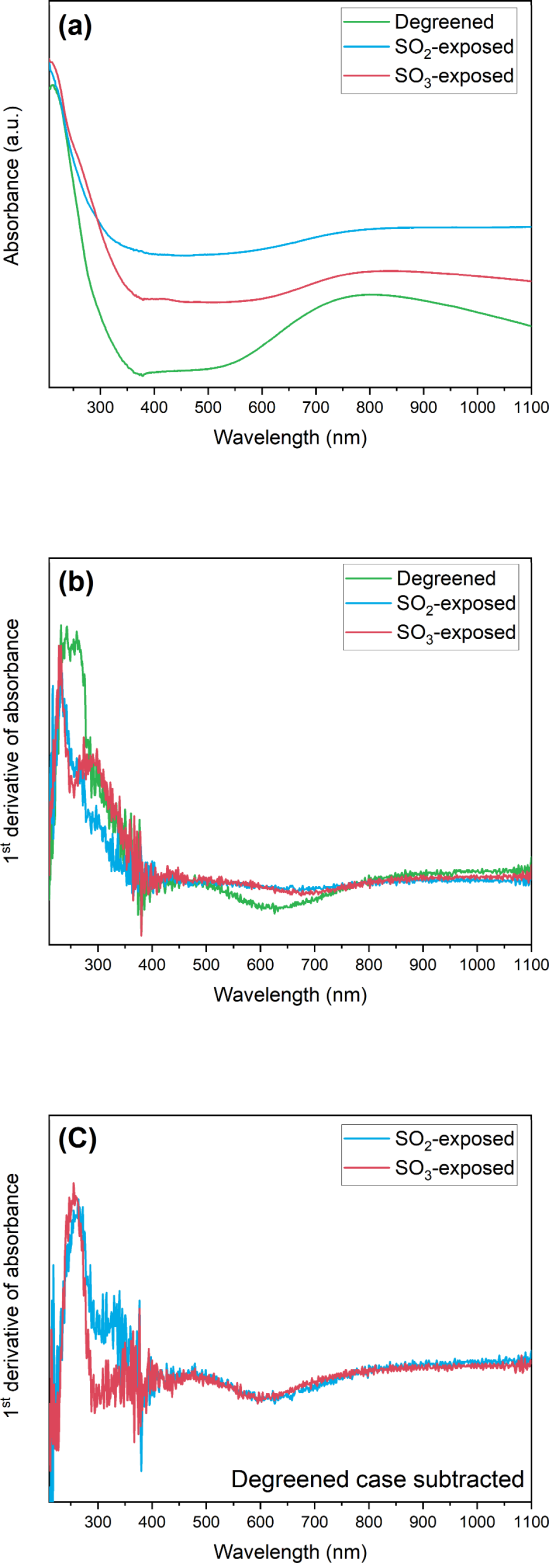
UV–visible diffuse reflectance spectra for degreened and SO_2_- and SO_3_-exposed powder samples in terms of (a) absorbance, (b) first derivative of absorbance, and (c) first derivative of absorbance with the degreened sample subtracted.

UV–visible diffuse reflectance spectroscopy (UV–vis-DRS) was performed to investigate possible formation of different sulfur species. Reference UV spectra were measured for copper compounds (i.e., CuSO_4_, Cu(OH)_2_, CuO) and various ammonium (bi)sulfite/sulfate in hydrated and ambient conditions, as shown in Figure S5. Figure 4 presents the resulting UV spectra as a function of wavelength for degreened and SO_2_- and SO_3_-exposed samples. Figure 4a shows for all samples two typical UV absorption features corresponding to the d–d transition of hydrated Cu^2+^ ions (ca. at 800 nm) and the ligand to metal charge transfer (LMCT) transition caused by the isolated Cu^2+^ ions (lattice O → Cu^2+^, at ca. 220 nm) of Cu/SSZ-13, as reported in literature.^49,80,81^ Moreover, UV absorption was observed for both SO_2_- and SO_3_-exposed samples over the entire measured wavelength, which was not the case for the degreened sample. This suggests the presence of ammonium (bi)sulfite/sulfate species since these species display considerable UV absorbance over the entire wavelength, as shown in Figure S5b. Both SO_2_- and SO_3_-exposed samples show in addition a broader UV absorption band at 250–300 nm compared to the degreened sample. The first derivative of Figure 4a was applied to qualitatively examine the effect of SO_2_ and SO_3_ with NH_3_ oxidation as a middle step, as shown in Figure 4b. The result shows different features of the SO_2_- and SO_3_-exposed samples compared to the degreened sample, especially at 220–400 nm. Dissimilar UV absorption features between the SO_2_- and SO_3_-exposed samples as in Figure 4c indicates the possible formation of different types of sulfur species interacting with NH_3_.

In comparison with reference bulk materials in Figure S5, CuSO_4_ (Figure S5a) and various ammonium (bi)sulfites/sulfates (Figure S5b) were considered as potentially formed sulfur species arising during SO_2_ and SO_3_ exposure. In the region of 220–400 nm, the broad UV absorbance for CuSO_4_ and ammonium (bi)sulfite/sulfate indicates that copper sulfate can be potential sulfur species interacting with Cu^2+^ ions, and the observed additional absorbance (shoulder at 250–300 nm in Figure 4a) is likely to originate from ammonium sulfate ((NH_4_)_2_SO_4_) or ammonium sulfite ((NH_4_)_2_SO_3_). To exclude the background resulting from Cu-CHA with respect to the SO_2_- and SO_3_-exposed samples, the UV spectrum of the degreened sample was subtracted from the SO_2_- and SO_3_-exposed samples. In Figure 4c, the resulting first derivative of UV absorbance shows the comparison of the derived sulfur species during the SO_2_ and SO_3_ exposure with NH_3_ saturation. By comparing the reference samples (i.e., bulk ammonium (bi)sulfite/sulfate) in Figure S5d, the result implies that the SO_2_ and SO_3_ exposures, with NH_3_ oxidation as the middle step, are associated with the formation of ammonium sulfite ((NH_4_)_2_SO_3_), ammonium sulfate ((NH_4_)_2_SO_4_), or (bi)sulfate ((NH_4_)HSO_4_).

### 3.3 H_2_ TPR in Degreened and SO_2_- and SO_3_-Exposed Cu/SSZ-13

XRD and UV–vis-DRS results suggest that different sulfated species are interacting with NH_3_ and copper ions inside the CHA unit cell, resulting from SO_2_ and SO_3_ exposure of Cu-CHA (with ammonia oxidation as a middle step). This implies that the formed sulfated species can influence the reduction properties of isolated Cu^2+^ ions. Bergman et al.^60^ showed that SO_2_ exposure influences the reduction property of Cu^2+^ in Cu-CHA using in situ XAS. Thus, H_2_ temperature-programmed reduction (H_2_-TPR) was carried out to investigate the reduction property of the SO_2_- and SO_3_-exposed samples.

**Figure 5:**
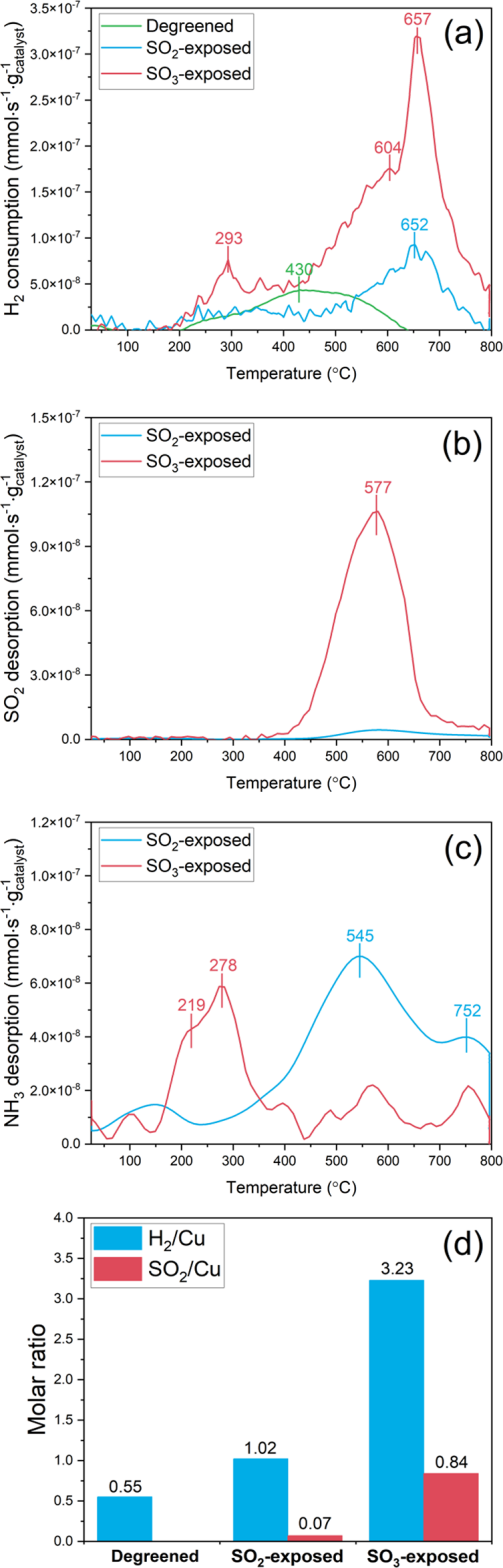
H_2_ temperature-programmed reduction for degreened and SO_2_- and SO_3_-exposed samples showing H_2_ consumption (a), desorption of SO_2_ (b), and NH_3_ release (c), and molar ratios of consumed H_2_ and desorbed SO_2_ per Cu content of samples (hydrated condition, gas feed: 0.2% H_2_/Ar, heating rate: 10 °C·min^–1^, total flow rate: 20 N mL·min^–1^).

Figure 5a shows H_2_ consumption as a function of temperature for degreened and SO_2_- and SO_3_-exposed samples. The degreened sample shows H_2_ consumption in the temperature range of 200–650 °C, resulting from Cu^2+^ species reduction by H_2_. In general, ZCuOH and Z_2_Cu are regarded as primarily isolated Cu^2+^ species over Cu-CHA and the observed H_2_ consumption peaks are commonly assigned to ZCuOH and Z_2_Cu at low and high temperatures, respectively, due to different binding strengths of Cu^2+^ species interacting with the CHA framework.^5,48,73,82,83^ In addition, in our recent study, combining ab initio calculations with experimental results, we also found the importance Z_2_CuOCu, Z_2_CuHOOHCu, and Z_2_CuOOCu to describe the reduction process.^84^

For the degreened sample in Figure 5a, the main reduction peak at 430 °C was impeded due to sulfur species, implying an interaction between sulfur and copper species. In contrast to the degreened sample, the sulfated samples show larger H_2_ consumption. SO_2_ and NH_3_ signals were detected as shown in Figure 5b,c, suggesting that SO_2_ and NH_3_ originated from decomposition of the remaining ammonium (bi)sulfite/sulfate species. The SO_2_-exposed sample shows two distinct reduction features during H_2_ TPR at ca. 293 and 652 °C (Figure 5a). For the SO_3_-exposed sample, relatively intense reduction peaks were observed at 657 °C with a shoulder (604 °C) and at 293 °C. The SO_3_-exposed sample showed three distinct H_2_ consumption peaks at 293, 604, and 657 °C, but only two reduction peaks corresponding to ca. 293 and 652 °C were observed for the SO_2_-exposed sample. Thus, it is likely that the peak at 293 °C resulted from hydrogen reacting with ammonium sulfite/sulfate species and the peaks at 604 and 657 °C originated from sulfur species interacting with Cu^2+^ ions since SO_2_ started to be observed above 400 °C with a peak at 577 °C in Figure 5b. In addition, an H_2_S (mass 34) was measured and a weak signal was detected above 650 °C for the SO_3_-exposed sample in Figure S6. Based on this, we suggest that the main hydrogen consumption peak at 657 °C might be associated with H_2_S formation since it requires a two-electron transfer to form H_2_S as well as cleavage of S–O/S=O bonds to become the sulfide (S^2–^) of H_2_S. It should be noted that the detection of H_2_S in the MS is rather difficult, and we suggest that this is the reason for the significantly smaller and noisier H_2_S peak compared to the hydrogen consumption peak. In contrast. no H_2_S signal was observed for the SO_2_-exposed sample. This is probably because of the lower amount of H_2_S formation as a result of the relatively lower sulfur content in the SO_2_-exposed sample compared to the SO_3_-exposed sample according to the ICP results in Section S3, SI. Both SO_2_- and SO_3_-exposed samples showed an SO_2_ desorption, with a single peak in the same temperature region (peak at 577 °C) (see Figure 5b). However, the sulfur release from the SO_3_-exposed sample was significantly larger. Note that H_2_SO_4_ and SO_3_ signals were not detected during H_2_-TPR but these compounds would be challenging to detect in the calorimeter setup if they are in low concentrations. In addition, ammonia desorption was clear from both SO_2_- and SO_3_-exposed samples, with two desorption peaks Figure 5c.

The consumed H_2_ per Cu and SO_2_ release per Cu from the H_2_ TPR are shown in Figure 5d. For the degreened sample without sulfur added, the H_2_/Cu ratio is 0.55, indicating that a one-step reduction of copper occurred. This is common at these temperatures, since a full reduction to metallic copper usually requires higher temperatures. Larger H_2_ consumption is found for the SO_2_-exposed sample, which is significantly increased for the SO_3_-exposed sample, simultaneously with the SO_2_ desorption increasing. Larger H_2_/Cu and SO_2_/Cu ratios are expected if the SOx-exposed sample involves a larger sulfur content. Figure 5d suggests that SO_3_ exposure results in a much larger sulfur uptake compared to SO_2_ exposure. This is in line with the ICP analysis results regarding the sulfur content (Section S3, SI). Considering the observed NH_3_ release and SO_2_ desorption features in Figure 5b,c, it can be concluded that NH_3_-sulfur interacting species remained following the sulfation and ammonia exposure step at 400 °C. Different sulfur speciation is expected to result from the SO_2_ and SO_3_ exposure, with NH_3_ as a middle step, forming ammonium sulfite ((NH_4_)_2_SO_3_) and ammonium sulfate ((NH_4_)_2_SO_4_) or (bi)sulfate ((NH_4_)HSO_4_) species according to the UV–vis DRS results.

### 3.4 AN-TPD with Degreened and SO_2_- and SO_3_-Exposed Cu/SSZ-13

AN can easily be formed from NO_2_ and NH_3_, and during its decomposition, it is well known that N_2_O is formed. Normally, a larger amount of N_2_O is reported as the NO_2_ concentration is increased during SCR reaction.^35^ We have reported increased N_2_O formation for Cu-CHA compared to H-CHA, indicating that AN interacts with copper species.^5^ It is, therefore, expected that SO_2_ and SO_3_ influence the AN and N_2_O formation.

**Figure 6:**
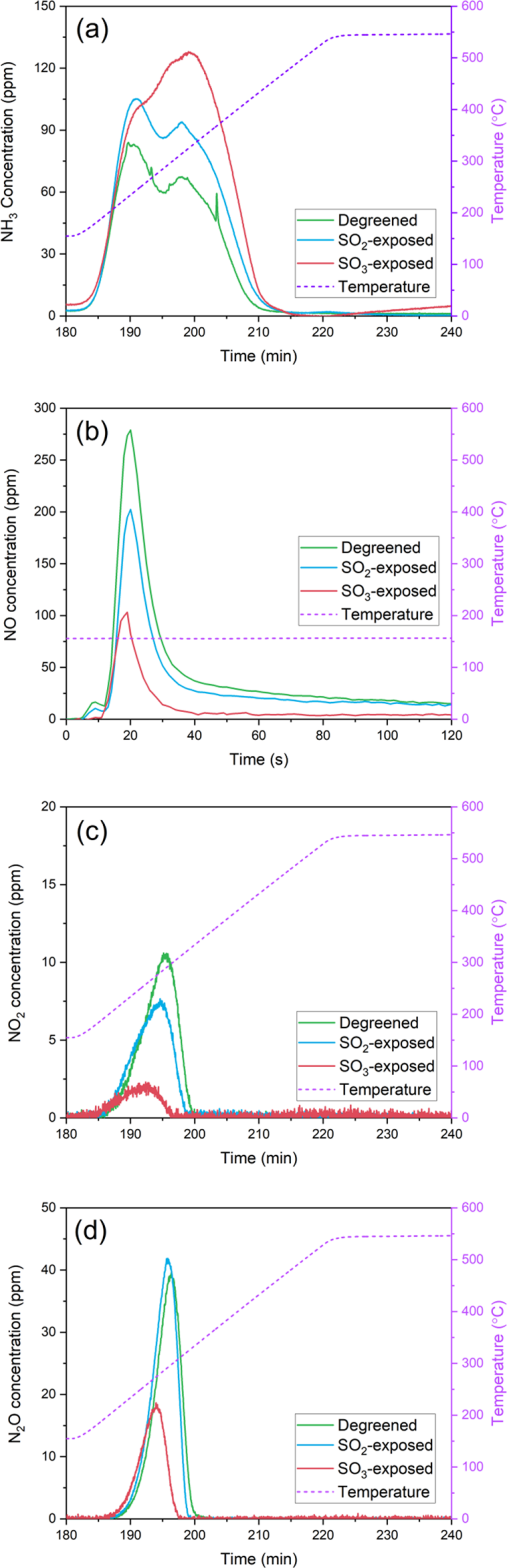
NH_3_ (a), NO (b), NO_2_ (c), and N_2_O (d) concentrations during ammonium nitrate temperature-programmed desorption for degreened and SO_2_- and SO_3_-exposed samples (gas feed: 200 ppm of NH_3_/NO_2_ + 5% H_2_O + Ar at 150 °C during adsorption, heating rate: 10 °C·min^–1^, total flow rate: 1200 N mL·min^–1^). Note that the NO concentration is during the adsorption phase.

Temperature-programmed desorption with AN (AN-TPD) was carried out for degreened and SO_2_- and SO_3_-exposed samples to investigate the effects of SO_2_ and SO_3_ on AN and N_2_O formation. The mass 28 (N_2_) signal was detected, while 200 ppm of NH_3_/NO_2_ was fed for 90 min in the empty-tube test (see Figure S7). This suggests that the NO_2_ SCR occurred during the ionization process in the mass spectrometer. In addition, no N_2_O signal was seen during heating when measuring with the FTIR as shown in Figure S7, suggesting that no AN was formed in the reactor system. The results for AN-TPD over Cu/SSZ-13 are presented in Figure 6. The NH_3_ concentrations during heating are presented in Figure 6a, where the NH_3_ release was increased for SO_2_- and SO_3_-exposed samples compared to the degreened samples. An increased NH_3_ release is expected due to residual sulfur species, since ammonium (bi)sulfite/sulfate species could form during the NH_3_+NO_2_ feed period of the AN-TPD test. These results are in line with the studies in the literature,^43,58,85,86^ where ammonia was suggested to interact with sulfate species resulting in larger ammonia storage. Notably, the SO_3_-exposed sample shows the largest NH_3_ release; furthermore, its NH_3_ release peak is also shifted from lower to higher temperatures compared to that for the SO_2_-exposed and degreened samples.

Figure 6b shows the NO formation curves during NH_3_+NO_2_ adsorption for degreened and SO_2_- and SO_3_- exposed samples. It has been reported that NO formation originates from NO_2_ disproportionation and subsequent surface nitrite oxidation by NO_2_, leading to surface nitrate formation with gas-phase NO release.^87−89^ The ratios for NO formed divided by removed NO_2_ during the adsorption phase were 0.03, 0.03, and 0.01 for the degreened, SO_2_-exposed, and SO_3_-exposed samples, respectively. A ratio of 0.33 is expected due to the disproportionation reaction where nitrates are formed.^89^ Thus, these values are significantly lower and the reason for this is likely that we also have slow NO_2_ SCR occurring at this temperature, since we use a quite high temperature (150 °C) and a large amount of the NO_2_ that is consumed is due to the SCR reaction. NO formation was decreased for both SO_2_-exposed and, in particular, SO_3_-exposed samples compared to the degreened sample. This suggests that surface nitrate storage was decreased due to sulfur species interacting with copper species since sulfur species do not show noticeable interaction with Brønsted sites.^45^ In this respect, it is postulated that most surface nitrate was formed on copper species under these conditions (presence of water, for example), since significantly reduced NO formation was observed for the SO_3_-exposed sample containing the largest sulfur content. Indeed, lower NO_2_ formation during the heating is found (Figure 6c), which is in line with the decreased surface nitrate storage, as NO_2_ formation is expected from the decomposition of the formed surface nitrate species during the NH_3_+NO_2_ adsorption.

Figure 6d shows N_2_O formation during heating for the degreened and SO_2_- and SO_3_-exposed samples. N_2_O formation due to thermal AN decomposition is commonly observed.^90^ The N_2_O curve from the SO_2_-exposed sample shifted toward slightly lower temperatures compared to the degreened sample. Moreover, a similar amount of N_2_O was released from the SO_2_-exposed sample (31.9 μmol·g_washcoat_^–1^) as for the degreened sample (31.3 μmol·g_washcoat_^–1^). It is slightly more N_2_O amount for the SO_2_-exposed sample (↑ 1.9%), but the differences could be within the accuracy of the experiments. Despite similar N_2_O formation, the NO_2_ release was clearly decreased for the SO_2_-exposed sample (Figure 6c) as compared to that of the degreened sample, which we interpret as a result of nitrate decomposition. Thus, these results overall indicate that AN formation also decreased for the SO_2_-exposed sample. However, it should be noted that some of the released NO_2_ could react in the slow NO_2_ SCR reaction. In contrast to the SO_2_-exposed sample, the SO_3_-exposed sample showed a remarkably decreased N_2_O release (14.6 μmol·g_washcoat_^–1^) corresponding to a ca. 53% reduction in N_2_O release compared to the degreened sample. The N_2_O peak was also shifted toward lower temperatures compared to the SO_2_-exposed and degreened samples. This suggests that SO_3_ exposure has a stronger influence on N_2_O formation compared to SO_2_ exposure.

It should be mentioned that sulfur species such as SO_2_, SO_3_, H_2_SO_4_, and H_2_S were measured by FTIR during AN-TPD. Among the sulfur species, SO_2_ and H_2_SO_4_ were observed but the SO_3_ and H_2_S were below the detection limit. However, a slightly negative SO_2_ signal (below 0 ppm) was observed from the SO_2_- and SO_3_-exposed samples that was symmetrical with the positive H_2_SO_4_ curve, as shown in Figure S8. Thus, it is likely that there were overlapping bands in the FTIR that were slightly overcompensated by the software for SO_2_ and H_2_SO_4_ according to Figure S9. To ease interpretation, the H_2_SO_4_ and SO_2_ signals were merged as SO_2_+H_2_SO_4_ (blue curve in Figure S8), and sulfur species are denoted in this paragraph. The desorption of these sulfur species features was still maintained, showing three desorption peaks at 250, 368, and 537 °C for the SO_2_-exposed sample and a monotonically increasing SO_2_+H_2_SO_4_ curve with a peak at 533 °C for the SO_3_-exposed sample. The marked different desorption features for these sulfur species suggests that SO_3_ exposure causes more stable sulfur species compared to SO_2_ exposure of Cu-CHA. For the SO_2_-exposed sample, the H_2_SO_4_ peaks at 250 and 368 °C were located at same positions with two NH_3_ release peaks at 180–215 min, as shown in Figure 6a. On the other hand, the SO_3_-exposed sample showed an even larger NH_3_ release; however, there was no significant desorption of sulfur species during NH_3_ release as compared to the SO_2_-exposed sample.

### 3.5 In Situ DRIFTS Measurement with Degreened and SO_2_- and SO_3_-Exposed Cu-CHA

AN-TPD results showed decreased NO formation during the NH_3_+NO_2_ feed for the SO_2_- and SO_3_-exposed samples, implying that surface nitrite oxidation with NO_2_ was severely limited. Especially, the SO_3_-exposed sample led to a considerably lower N_2_O formation. However, the SO_2_-exposed sample gave comparable N_2_O formation compared to the degreened sample, although less NO_2_ formation was observed, consistent with that less AN was formed, as evident from the NO formation in the adsorption phase. To corroborate on these results, surface nitrite/nitrate species were investigated with DRIFTS in the presence of 400 ppm of NO_2_ + 1% H_2_O + Ar at 150 °C. It should be mentioned that a relatively higher concentration of H_2_O is usually introduced to mimic the real exhaust environment. However, 1% H_2_O was set as the base feed here along with the background feed (Ar) since this was the maximum water feed in our DRIFTS setup. In addition, note that the powder sample was used for the degreened case, while crushed monoliths were used for the SOx-exposed monoliths. Thus, the sulfur exposed samples contain a large amount of cordierite and the spectra were therefore normalized with the amount of copper they contained.

**Figure 7:**
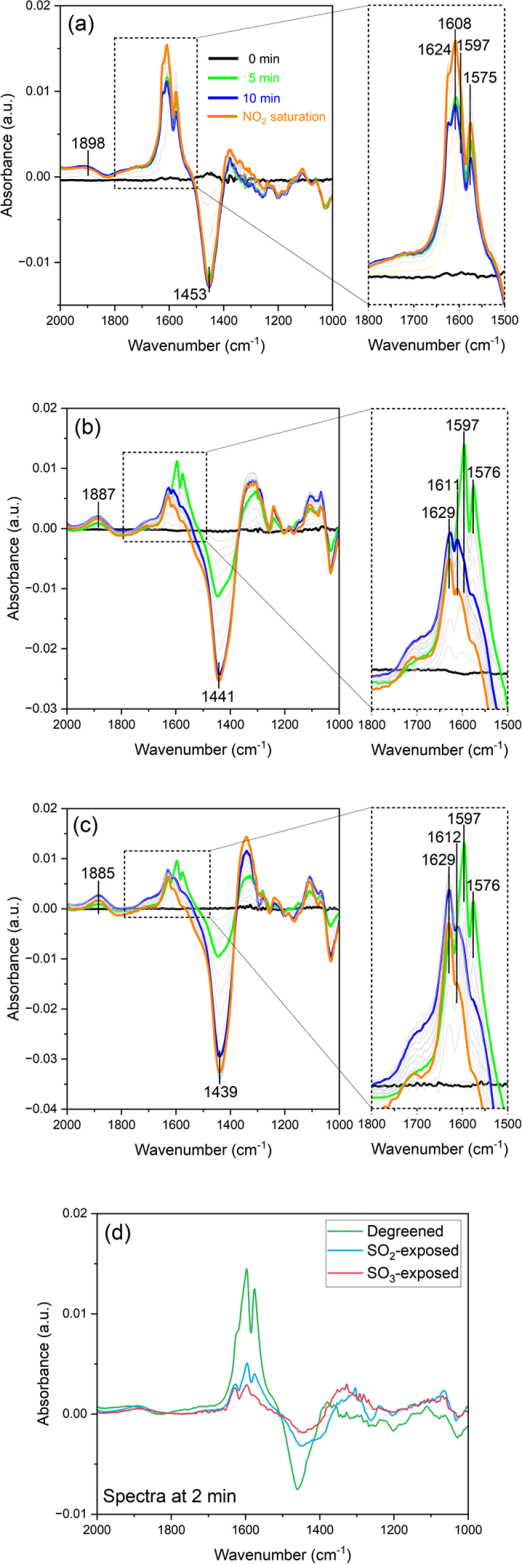
Spectrum evolution during NO_2_ adsorption in DRIFTS for the (a) degreened, (b) SO_2_-exposed, and (c) SO_3_-exposed samples with (d) comparison of the NO_2_ spectra of panels (a), (b), and (c) after 2 min of NO_2_+H_2_O exposure. Spectral evolution with gray represents intermediate spectra prior to NO_2_ saturation (orange). Spectra in all panels were normalized by the number of moles of copper within the sample cup (gas feed: 400 ppm of NO_2_ + 1% H_2_O + Ar at 150 °C, total flow rate: 100 N mL·min^–1^, sample bed temp.: 150 °C).

Figure 7 shows the NO_2_ adsorption phase for the degreened AND SO_2_- and SO_3_-exposed samples at 150 °C. The resulting spectral evolutions are illustrated in the region 2000–1000 cm^–1^ to investigate the surface adsorption species. The surface nitrate region (1800–1500 cm^–1^) is magnified in the right panel from the original spectrum marked with the dashed line box. The addition of 400 ppm of the NO_2_ + base feed mixture (Figure 7a) results in peaks related to the formation of chelating bidentate ZCu^2+^-(NO_3_) structures (1750–1500 cm^–1^).^91^ The four bands at 1624, 1608, 1597, and 1575 cm^–1^ are associated with υ(NO) vibrations of different types of chelating bidentate nitrates.^91,92^ The peak at 1608 cm^–1^ reached the highest intensity, when NO_2_ was saturated (orange curve). Typical NO^+^ formation (∼2155 cm^–1^)^93^ was not observed here under an excess of water, due to H_2_O interaction with Brønsted sites Si(OH)Al. The negative band (∼1453 cm^–1^) is associated with H_2_O removal.^94^ The band 1898 cm^–1^ was assigned as NO interaction with Cu^2+^ (i.e., Cu^2+^-NO).^95^ Note that the negative peak (∼1453 cm^–1^) may affect the neighboring nitrate feature.

For the SO_2_-exposed sample in Figure 7b, surface nitrate features were observed over the region 2000–1000 cm^–1^. The four bands associated with nitrate species (1629, 1611, 1597, and 1576 cm^–1^) were developed within 5 min and then consumed. Moreover, bands 1887 (positive band) and 1441 cm^–1^ (negative band) were observed as well as the degreened sample. Surprisingly, at 10 min (blue curve), a decrease in the surface nitrate species peaks was observed, which were further decreased until NO_2_ saturation (orange curve). During NO_2_ adsorption, a decrease in the nitrates is not expected. The negative band at 1441 cm^–1^, due to water removal,^94^ is quite large. It is possible that there is an overlap between the 1441 cm^–1^ negative band and the nitrate bands; thus, when the water removal increases, it decreases the bands in the nitrate region. This is not observed for the degreened sample, and the reason for that could be that the nitrate peaks are significantly larger; thus, the interference with the band at 1441 cm^–1^ might be less. For the SO_3_-exposed sample in Figure 7c, a similar trend was observed as the SO_2_-exposed sample. For example, the surface nitrate region increased at 5 min (green curve), followed by decreased intensities until NO_2_ saturation (orange curve). Likewise, for the degreened sample, the positive band at 1885 and negative band at 1439 cm^–1^ were observed. In Figure 7d, a comparison is done between the samples after only 2 min of NO_2_+H_2_O exposure, where the water peaks for the SO_2_- and SO_3_-exposed samples are still quite small. From these results, it is clear that the nitrate bands are decreased for SO_2_- and SO_3_-exposed samples compared to the degreened sample.

To summarize, the DRIFT results show that there are fewer nitrates on the SO_2_- and SO_3_-exposed samples, as would be expected from the flow reactor experiments. However, it should be mentioned that there could be an overlap with the band for water removal, which could decrease the peak for nitrates.

## 4 Discussion

At low temperature, surface nitrate formation is observed in the presence of either NO+O_2_ or NO_2_ in Cu-CHA.^89,91,92,96^ An in situ DRIFTS measurement shows that NO_2_ kinetically promotes surface nitrate formation in Cu-CHA compared to NO+O_2_ presence in dry conditions.^89^ In wet conditions, the nitrate species formation is significantly hindered due to water interactions.^89^ NO release is typically seen during isothermal NO_2_ adsorption in Cu-CHA, as shown in Figure 6b as well as in the literature,^87,88,97,98^ resulting from surface nitrite oxidation by NO_2_, and thereby, NO is formed through the following reactions (R.1 and R.2) below. According to Ruggeri et al.,^89^ when H_2_O is present, Cu dimers are likely dissociated into two ZCuOH via R.3.
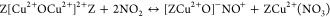
R.1
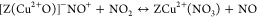
R.2
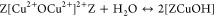
R.3

Ruggeri et al.^89^ derived R.1–R.3 by NO or NO_2_ exposure to Cu-CHA at 120 or 150 °C in dry or wet conditions using in situ DRIFTS. Cu dimers in R.1 are critically important as a surface oxygen provider, initiating surface nitrite formation and leading to the additional nitrate in the form of copper nitrate and NO via R.2. A recent study performed by Negri et al.^96^ reported that possible structures of nitrate species and their local environment show that only ZCuOH in the 8-MR within the CHA cage is the active copper species forming the framework-interacting chelating bidentate nitrate (i.e., Z[Cu^2+^(NO_3_)]).^96^ Other nitrate candidates such as bridging bidentate or monodentate nitrate configurations were difficult to reconcile the experimental features under exposure of Cu-CHA to an NO+O_2_ mixture.^91,96^

The SO_2_ and SO_3_ exposure to Cu-CHA significantly influences the surface nitrate formation. In the AN TPD (Figure 6b), the NO release during isothermal NH_3_+NO_2_ adsorption is significantly decreased. Since NO production during NO_2_ exposure is associated with nitrate formation, according to reactions R.1 and R.2,^89^ these results suggest that the surface nitrate formation is hindered due to resulting sulfur species after SO_2_ and SO_3_ treatment of Cu-CHA. The lowest NO release was observed for the SO_3_-exposed sample (see Figure 6b), suggesting that this sample exhibited the lowest amount of nitrates, which is consistent with the fact that it contained the largest amount of sulfur, as seen in the ICP measurements (Section S3, SI). These results are also consistent with the in situ DRIFT experiments (see Figure 7d). Our first-principles calculations shown in Figure 1b,d describe that the SO_3_ interaction with both Z_2_Cu and ZCuOH is possible. These findings are in agreement with ICP analysis showing an S/Cu molar ratio of 1.2, which indicates that all copper sites were occupied by sulfur species. In contrast, SO_2_ primarily interacts with ZCuOH but not Z_2_Cu as suggested by first-principles calculations (Figure 1a,c). Since ICP results show low sulfur storage for the SO_2_-exposed sample, it suggests that there is a low amount of ZCuOH in our sample. These results are consistent with our H_2_ TPR (Figure 5a) where the lower reduction peak, usually assigned to ZCuOH, is small for the degreened sample. A deconvolution of hydrogen TPR suggests only 15% ZCuOH. This value is slightly lower than the theoretical value according to Paolucci et al.,^26^ which is 35%. The reason for this could be that in our synthesis of SSZ-13, we use Na cations and, according to Lee et al.,^99^ SSZ-13 synthesis with the Na cation can increase 2Al sites over CHA. In our study, SSZ-13 was synthesized using Na and TMAda^+^, and this could result in a larger amount of 2Al, which increases Z2Cu and thereby decreases ZCuOH.

In terms of speciation of sulfur according to the first-principles calculation in Figures 1 and 2, SO_2_ interaction with ZCuOH results in (bi)sulfite (ZCuHSO_3_) and SO_2_ interactions with dicopper cause both (bi)sulfite and (bi)sulfate surface species. Meanwhile, the interaction of SO_3_ with Z_2_Cu and ZCuOH leads to only (bi)sulfate (ZCuHSO_4_). The (bi)sulfate resulting from the SO_3_ exposure shows a lower energy compared to the other sulfur species (i.e., ZCuHSO_3_) derived from the SO_2_ exposure. Importantly, SO_2_ and SO_3_ interaction with Cu dimers shows a further decrease in energy compared to the SO_2_ and SO_3_ adsorption with monomeric copper species (i.e., Z_2_Cu and ZCuOH). The difference between the interaction of SO_2_ and SO_3_ with Cu dimers is that SO_2_ adsorption causes both ZCuHSO_3_ and ZCuHSO_4_. The SO_3_ adsorption, however, results in only two ZCuHSO_4_. In this respect, two H_2_ consumption peaks at 604 and 657 °C can be associated with (bi)sulfate interacting with copper monomers and dimers, respectively, in Figure 5a.

As mentioned above and described in reaction R.3, in the presence of water, ZCuOH is the active species for the surface nitrate formation in the form of copper nitrate (ZCuNO_3_) by following reactions below (R.4–R.6), as described in the refs (89,100). In the feed of only NO_2_, NO^+^ on the copper site results from NO_2_ disproportionation with forming nitric acid (HNO_3_) via R.4. In R.6, the resulting copper-nitrite oxidation by NO_2_ leads to copper nitrate and NO. For the SO_3_-exposed sample, most copper sites are occupied by (bi)sulfate in the form of copper-(bi)sulfate (ZCuHSO_4_). ZCuHSO_4_, therefore, deactivates the surface nitrate formation by blocking the copper site from the access of the NO^+^ or HNO_3_, resulting in the lowest NO release during the adsorption phase in AN-TPD. In the same manner, decreased NO release was seen for the SO_2_-exposed sample compared with the degreened sample. However, more NO release was observed due to less sulfur content compared to the SO_3_-exposed sample.
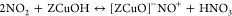
R.4
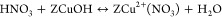
R.5
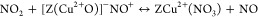
R.6

In AN-TPD, stored surface nitrate species are typically desorbed in the form of NO_2_ for the degreened sample, as can be seen in Figure 6c. For the SO_2_- and SO_3_-exposed samples, decreased NO_2_ release was seen because of decreased nitrate storage resulting from sulfur species, especially the SO_3_-exposed sample. With respect to N_2_O, the SO_3_-exposed sample released the lowest amount of N_2_O (14.6 μmol·g_washcoat_^–1^) compared to the SO_2_-exposed sample (31.9 μmol·g_washcoat_^–1^).

Surprisingly, a comparable amount of N_2_O was released for the SO_2_-exposed sample as degreened sample (31.3 μmol·g_washcoat_^–1^), although decreased nitrate storage was clearly observed via NO formation in Figure 6b. This suggests that sulfur may increase N_2_O selectivity during NO_2_ SCR. Sulfur enhances ammonia storage on the catalyst. We hypothesize that the increased availability of ammonia on the catalyst for the SO_2_ poisoned sample could result in that more N_2_O is formed compared to NO_2_ release. According to first-principle calculations performed by Feng et al.,^36^ the decomposition of the reaction intermediate (H_2_NNO) over the Cu site leads to N_2_O and H_2_O. Their suggested specific copper-configuration is NH_3_-solvated Cu dimers. In Figure S8, the observed SO_2_+H_2_SO_4_ release and N_2_O production (Figure 6d) show that for the SO_2_ poisoned sample, there is sulfur release already from 200 °C. The main N_2_O desorption occurs at 300 °C, suggesting that some of the copper sites were regenerated for the SO_2_-exposed sample; thus, resulting H_2_NNO intermediates can possibly lead to N_2_O through the regenerated copper sites. This combination with the increased availability of ammonia could be one possible explanation. However, for the SO_3_-exposed sample, there was significantly more sulfur stored on the sample as seen in ICP experiments. In addition, the sulfate species were very stable, which is seen by the fact that the SO_2_+H_2_SO_4_ release occurred at significantly higher temperature and was not detected during N_2_O formation. These results suggest that more copper sites were occupied by sulfur species when N_2_O was formed and in addition significantly less nitrates were formed in the adsorption phase, which could explain the low N_2_O formation for the SO_3_-exposed sample.

## 5 Conclusions

NO_2_+NH_3_ titration to form AN and subsequent temperature-programmed desorption was applied for the degreened and SO_2_- and SO_3_-exposed Cu-CHA to investigate the effect of SO_2_ and SO_3_ on surface nitrate and N_2_O formation. Sulfation was carried out at 400 °C to mimic the scenario of SO_2_/SO_3_ contamination of the SCR catalyst resulting from SO_2_ oxidation to SO_3_/H_2_SO_4_ over the DOC or coated filter. H_2_-TPR and in situ DRIFTS were applied to investigate the reduction properties and surface species response in the presence of sulfur species derived by SO_2_- and SO_3_-exposure treatments. Critical effects of SO_2_ and SO_3_ on Cu-CHA, its surface nitrate speciation, and N_2_O formation were found. The following major findings were established:

1. First-principles calculations demonstrate that SO_2_ adsorption on monocopper sites results in (bi)sulfite and dicopper causes both (bi)sulfite and (bi)sulfate surface species. SO_3_ adsorption on mono- and dicopper results in only (bi)sulfate. Sulfur species derived from the SO_3_ exposure are lower in energy compared to sulfur species derived from the SO_2_ exposure. In addition, SOx adsorption on the dicopper exhibits higher thermodynamic stability as compared to SOx adsorption on the monocopper in Cu-CHA, indicating that speciation of copper has a significant effect on the speciation and thermal stability of sulfur species over Cu-CHA.
2. SO_3_ exposure causes a more detrimental contamination of Cu-CHA compared to SO_2_ exposure, according to the ICP and H_2_-TPR results, in line with an earlier reported SO_2_/SO_3_-exposure study^9^ as well as showing good agreement with our theoretical calculations. The SO_2_ exposure mainly shows sulfur contamination with ZCuOH, but the SO_3_ exposure results in the sulfation not only with ZCuOH but also with Z_2_Cu. Therefore, the SO_3_-exposed Cu-CHA shows a larger sulfur uptake compared to the SO_2_-exposed Cu-CHA.
3. In situ DRIFTS demonstrated that similar surface species result from reactions between NO_2_ and sulfur species derived from the SO_2_ and SO_3_ exposures. After 2 min of NO_2_ exposure, significantly lower bands were observed in the nitrate region for the SO_2_- and SO_3_-exposed samples compared to the degreened sample. The results from DRIFT could explain why NO release was decreased for both SO_2_- and SO_3_-exposed samples during NH_3_+NO_2_ adsorption in AN-TPD. Especially, the SO_3_-exposed sample showed the lowest NO formation, suggesting that surface nitrate formation was significantly hindered due to (bi)sulfate occupying the copper sites in the form of copper-(bi)sulfate (ZCuHSO_4_).
4. AN-TPD showed similar N_2_O release for the SO_2_-exposed Cu-CHA compared to the degreened Cu-CHA, while a lower NO_2_ release for SO_2_-exposed Cu-CHA. These results suggest that SO_2_ exposure can increase the N_2_O selectivity compared to the degree end Cu-CHA during AN decomposition. In contrast, SO_3_-exposed Cu-CHA shows significantly lower N_2_O formation compared to the degreened and SO_2_-exposed Cu-CHA, due to its highly stable sulfur species (i.e., Cu-(bi)sulfate). SO_3_ exposure caused more stable sulfur species compared to SO_2_ exposure. The stability and amount of the sulfur species are likely important factors that influence N_2_O and N_2_ selectivity since SO_2_-exposed and SO_3_-exposed samples shows totally different N_2_O formation properties.

## Supporting Information Available

Hydrothermal synthesis of Cu-CHA and washcoating on the monolith substrate, SO_2_ and SO_3_ exposure to the Cu-CHA procedure with SO_3_ calibration, elemental analysis results from ICP, N_2_ physisorption and XRD, SEM images of zeolite crystal morphology, UV–vis DRS measurement for bulk sulfur-containing compounds, H_2_S detection during H_2_-TPR, DRIFTS, empty-tube test during the adsorption step, and SO_2_/H_2_SO_4_/N_2_ release during heating in AN-TPD (PDF)
