# Supplementary material for: Effect of SO2 and SO3 Exposure to Cu-CHA on Surface Nitrate and N2O Formation for NH3–SCR
Source: ACS Eng Au. 2024 May 31;4(4):405–21. doi: 10.1021/acsengineeringau.4c00004 (PMC11342297; doi:10.1021/acsengineeringau.4c00004)
Supplement: Supplementary file 1 — eg4c00004_si_001.pdf [file eg4c00004_si_001.pdf]

Supplementary Information

Effect of SO_2 and SO_3 exposure to Cu-CHA on surface nitrate and N_2O formation for NH_3-SCR

Joonsoo Han[a]*, Joachim D. Bjerregaard[b], Henrik Grönbeck[b], Derek Creaser[a] and Louise Olsson[a]*

[a]Department of Chemistry and Chemical Engineering, Competence Centre for Catalysis, Chalmers University of Technology, 41296 Göteborg, Sweden

[b]Department of Physics and Competence Centre for Catalysis, Chalmers University of Technology, 41296 Göteborg, Sweden

Contents

S1. Catalyst synthesis and monolith preparation

S2. SO_3 calibration and SO_x-exposure of degreened monoliths

S3. Elemental analysis results from ICP and N_2 physisorption

S4. UV visible diffuse reflectance spectroscopy (UV-Vis.-DRS)

S5. H_2 temperature programmed reduction (H_2-TPR)

S6. AN temperature programmed desorption (AN-TPD)

S7. In-situ diffuse reflectance infrared Fourier transform spectroscopy (DRIFTS)

*Corresponding author: E-mail: joonsoo@chalmers.se; louise.olsson@chalmers.se

S1. Catalyst synthesis and monolith preparation

Cu/SSZ-13 was prepared by following the reported synthesis methods in our previous study [1]. The Na form of SSZ-13 was synthesized to acquire a Si to Al molar ratio of ≈ 15. The prepared Na/SSZ-13 powder was transferred to NH_4 form via ion-exchange with a 0.1 M NH_4NO_3 solution. The obtained NH_4/SSZ-13 powder was washed several times with milli-Q water (18.2 MΩ·cm) until pH ≈ 7 was reached. Afterwards, the resulting powder was dried at 80℃ in a drying oven overnight and well ground as fine powder form to calcine the NH_4/SSZ-13 powder at 500℃ for 8 h in static air with a 2 ℃·min^{-1} heating rate to acquire the H form of SSZ-13. Finally, copper-exchanged SSZ-13 was prepared via an incipient wetness impregnation method with $Cu(NO_3)_2$ solution (0.074g of $Cu(NO_3)_2 \cdot 2.5H_2O$ + 0.35g of ethanol). The copper-exchanged solution was dried at room temperature overnight and then calcined in a calcination oven at 600℃ and 750℃ for 8 h and 6 h, respectively with a 2 ℃·min^{-1} heating rate.

Catalyst monoliths were prepared with honeycomb monolith substrates (cordierite, 400 cpsi, 15mm x 20 mm). The acquired Cu/SSZ-13 sample powders were washcoated onto the monolith substrates by dipping the monolith substrate into a mixture composed of powders substances (5 wt.% Boehmite binder + 95 wt.% Cu/SSZ-13 powder) in liquid (50 wt. % ethanol + 50 wt. % Milli-Q Water). The washcoated monoliths were carefully dried with a heating gun. The washcoating was repeated to obtain the target washcoat loading (ca. 300mg). Afterwards, the dried catalyst sample monoliths were calcined at 500℃ for 2h, with 2 ℃·min^{-1} heating rate.

S2. SO_3 calibration and SO_x-poisoning of degreened monoliths

Prior to SO_x treatments of the catalyst sample monoliths, SO_3 formation was calibrated by using an oxidation catalyst (Pt/Al_2O_3, 7.5 wt.% Pt). The temperature was set at 550℃ while 30 ppm SO_2 + 8% O_2 + Ar was fed to the oxidation catalyst. 30 ppm SO_2 and 200 ppm SO_2 concentrations were tested to confirm SO_3 formation, and the test results showed that SO_3 was generated and SO_2 oxidation reached its equilibrium state as shown in Figure S1. Afterwards, the SO_3 generation test was repeated several times to ensure reproducibility of the SO_3 formation via the oxidation catalyst for the SO_2+SO_3 treatment.

SO_2 and SO_2+SO_3 treatments were performed with the catalyst sample monoliths by the following test procedures illustrated in Figure S2. First, the fresh Cu/SSZ-13 monoliths were degreened under standard SCR conditions (400 ppm NH_3/NO + 10% O_2 + 5% H_2O + Ar Bal.) at 750℃ for 5 h,

following by SO_2 or SO_2+SO_3 treatment steps referred to as SO_2- and SO_3-poisoning at 400°C, respectively. Gas compositions are specified with coloured region as degreening, SOx feed, NH_3 feed, and base feed steps.

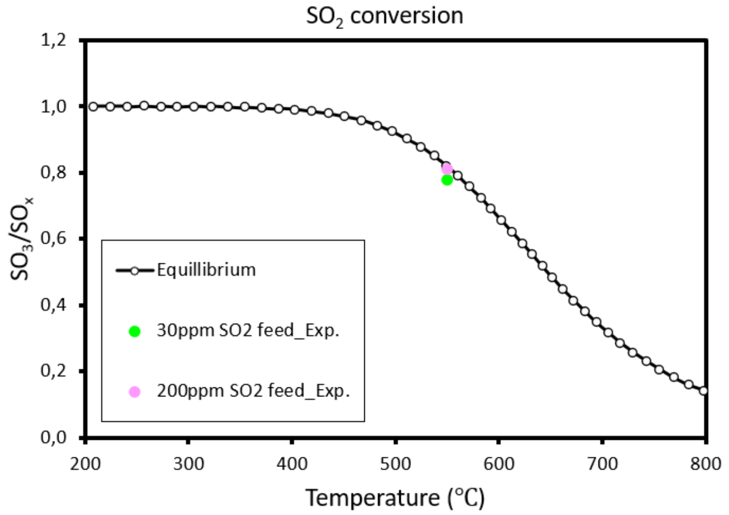

Figure S1. SO_2 conversion under dry conditions over Pt/Al_2O_3 catalyst in SO_3 generator upstream from SGB reactor. The equilibrium curve was computed according to the thermodynamic data obtained from NIST Chemistry Webbook [2].

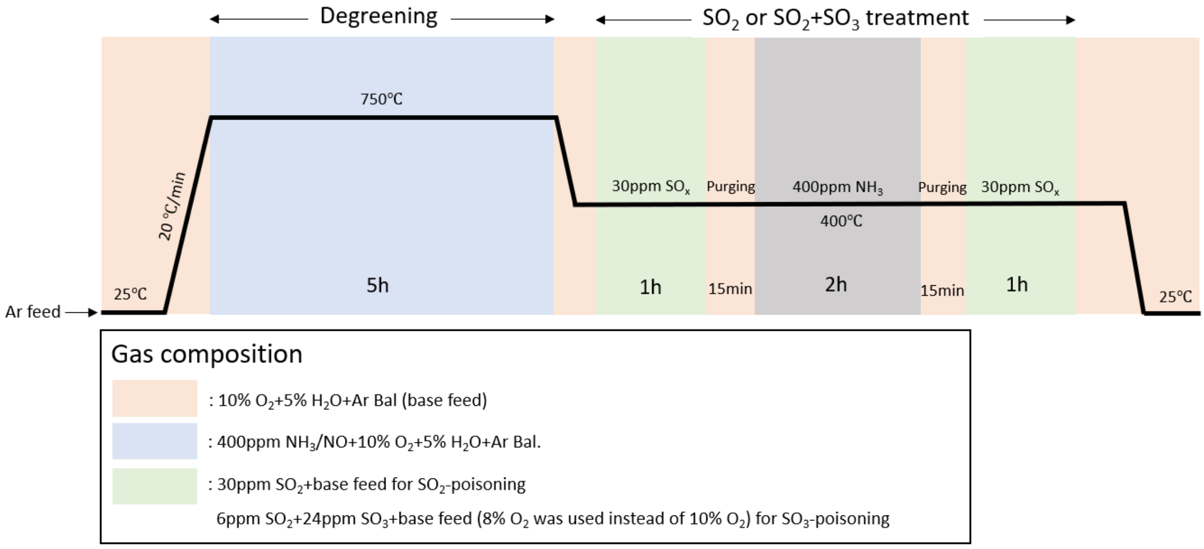

Figure S2. SO_2 and SO_2+SO_3 treatments of monoliths in the SGB reactor. The SO_2+SO_3 mixture was produced from an oxidation catalyst in a separate reactor prior to the main reactor. The gas also consisted of 8% O_2 + 5% H_2O + Ar fed with a total flow of 1200 Nml·min^{-1}.

S3. Elemental analysis results from ICP, N_2 physisorption and XRD

In terms of sulfated samples, the sulfated washcoated monoliths were well crushed to a very fine powder form. 0.25 and 1.22 of S/Cu molar ratio was obtained via ICP analysis, giving 33 and 159 μmol·$g_{washcoat}^{-1}$ of sulfur content for the SO_2- and SO_3-exposed sample, respectively.

Table S1. ICP and N_2 physisorption results of degreened H/SSZ-13 and Cu/SSZ-13.

Labeled sample	Sample	SAR[a]	Si [mg/kg]	Al [mg/kg]	Na [mg/kg]	Cu [mg/kg]	BET surface area[b] [m^2/g]
H-CHA	H/SSZ-13	13.8	344000	24000	<100	-	711
Cu-CHA	Cu/SSZ-13	13.5	335000	23900	<100	8300	651

[a] SAR indicates molar ratio of Si to Al.

[b] Specific surface area of the powder samples was measured with N_2 sorption using BET method.

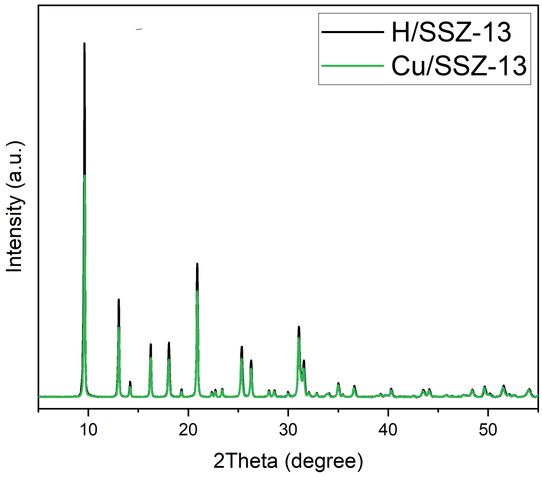

Figure S3. X-ray diffractogram for degreened H/SSZ-13 and Cu/SSZ-13 powders.

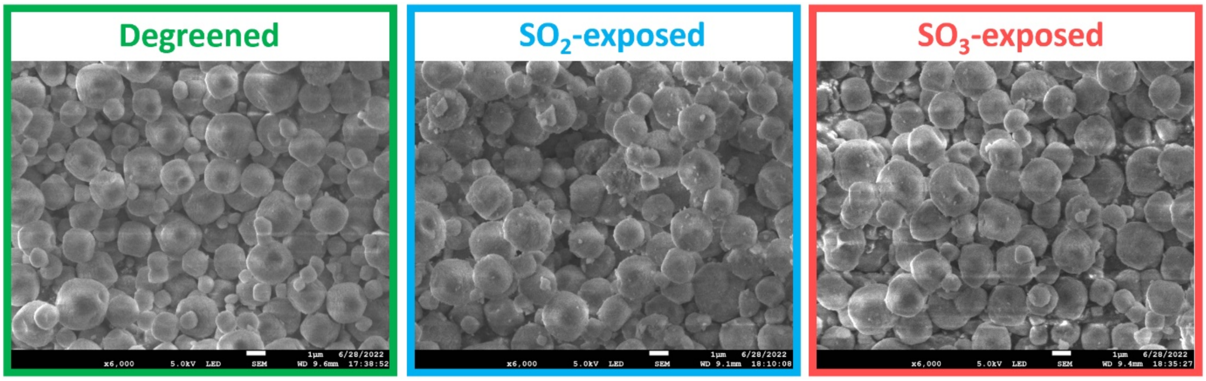

Figure S4. SEM images of zeolite crystal morphology for the degreened, SO_2-, and SO_3-exposed Cu-CHA. (Magnification: x 6000, applied voltage: 5 kV)

S4. UV visible diffuse reflectance spectroscopy (UV-Vis.-DRS)

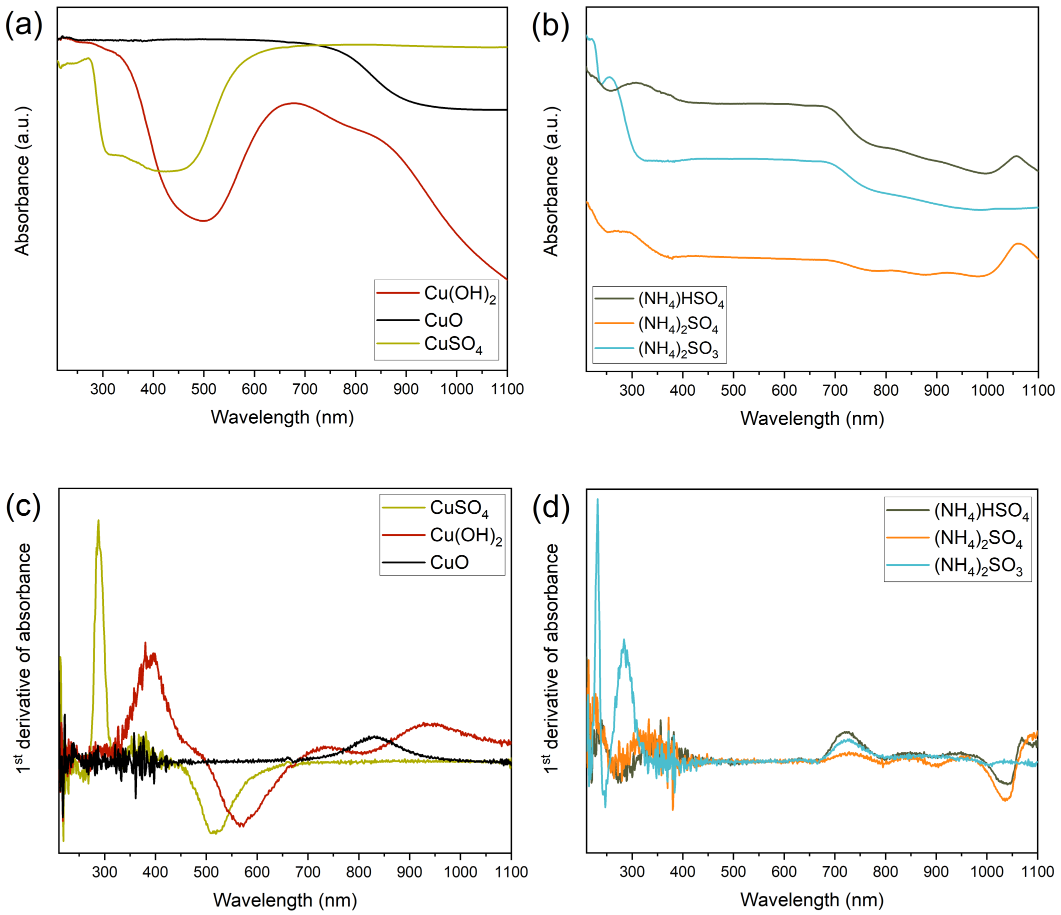

Figure S5. UV visible diffuse reflectance spectra on reference materials with copper compounds $Cu(OH)_2$, CuO, $CuSO_4$ (a), ammonium (bi)sulfate/sulfite (b), 1st derivative of resulted UV spectrum from copper compounds (c) and ammonium (bi)sulfate/sulfite (d).

S5. **H_2 temperature programmed reduction (H_2-TPR)**

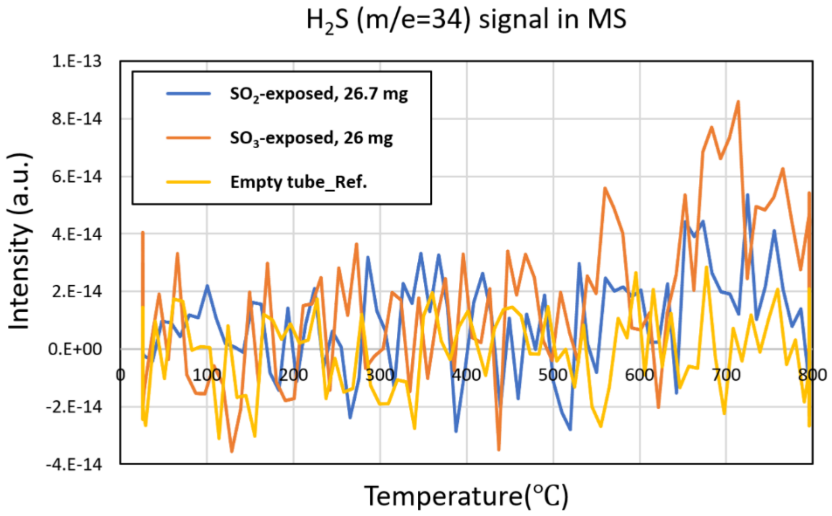

Figure S6. Mass 34 signal as function of temperature under 2000 ppm H_2/Ar feed during H_2-TPR. (heating rate: 10 ℃·min^{-1}, Total flow rate: 20 Nml·min^{-1}). Legend index: SO_2-exposed sample with blue (—), SO_3-exposed sample with orange (—), and empty tube with yellow (—).

S6. AN temperature programmed desorption (AN-TPD)

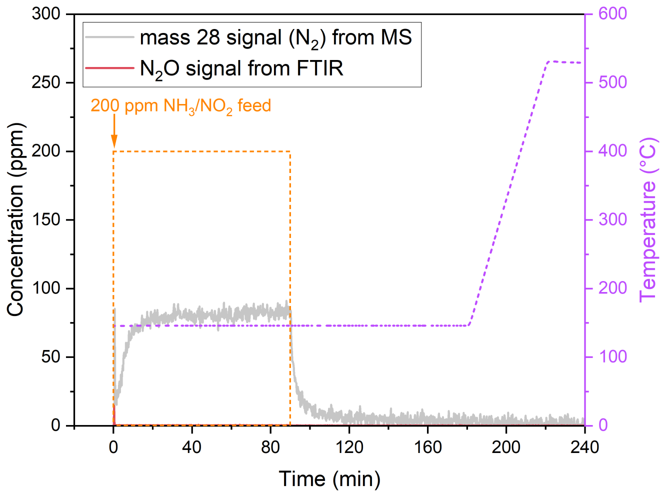

Figure S7. N_2 and N_2O signal from MS and FTIR in AN-TPD test with empty-tube.

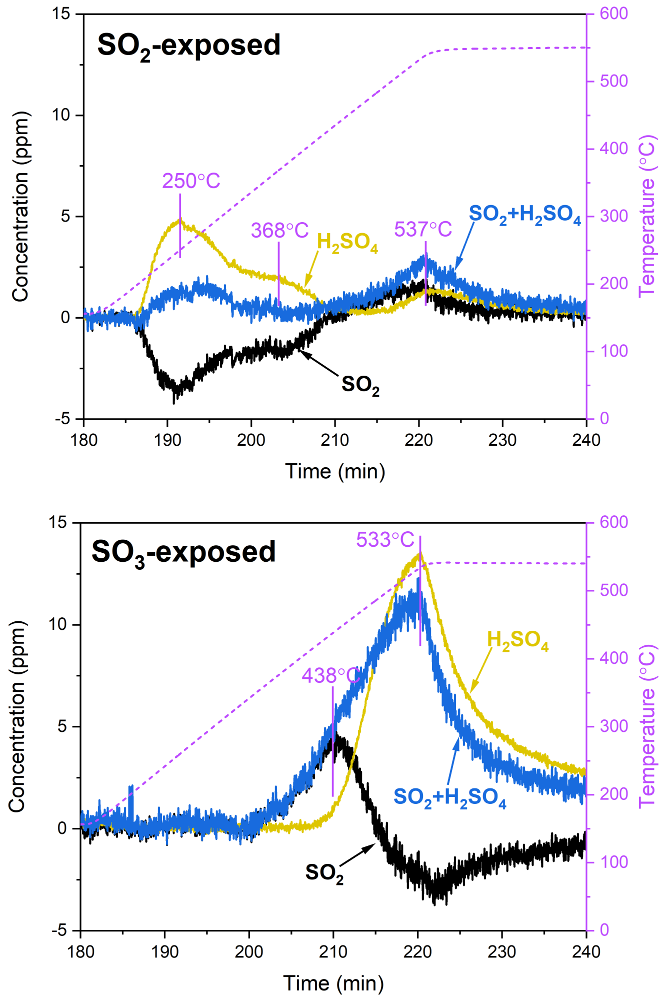

Figure S8. SO_2 and H_2SO_4 concentration during heating in AN-TPD test for SO_2- and SO_3-exposed samples.

(a) Sulfur dioxide (SO_2)

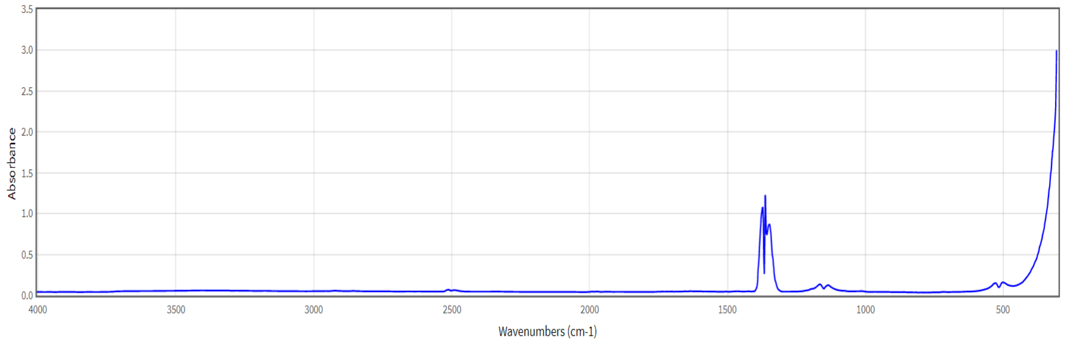

(b) Sulfuric acid (H_2SO_4)

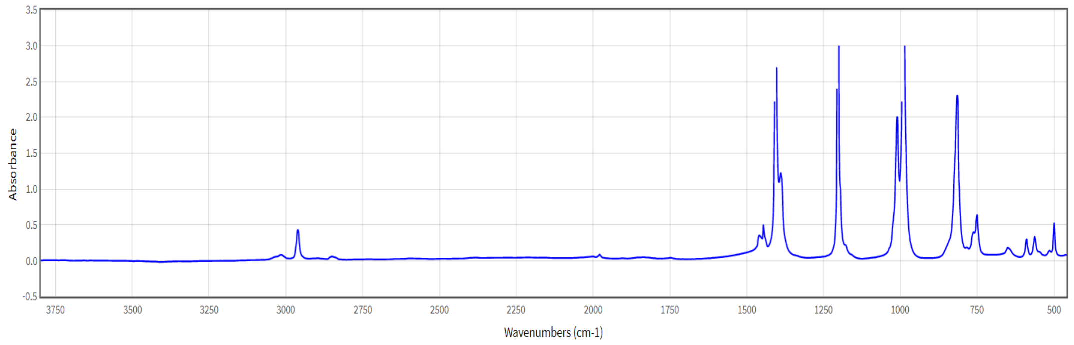

Figure S9. Infrared spectrum of SO_2 (a) and H_2SO_4 (b). These reference IR spectrums were taken from the data base in NIST Chemistry Webbook [2]

S7. In-situ diffuse reflectance infrared Fourier transform spectroscopy (DRIFTS)

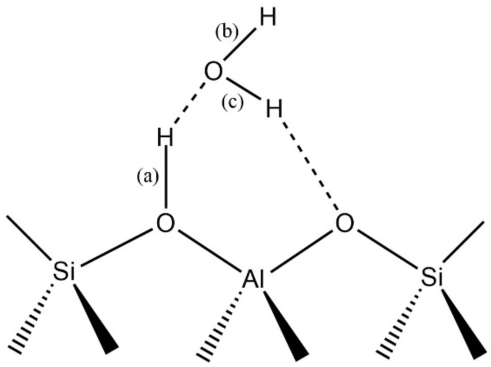

Scheme S1. Schematic illustration of Brønsted site interacting with an H_2O molecule [3]. Note that the origin of the proton is out of the focus of this study.

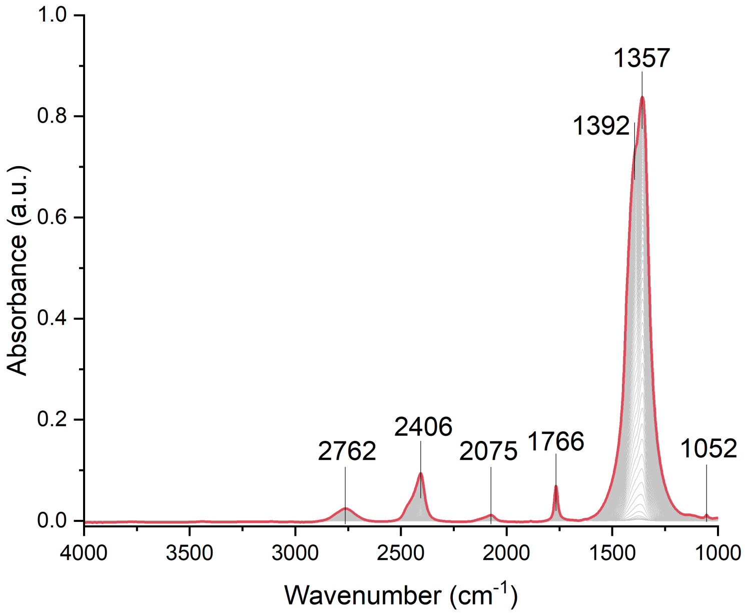

Figure S10. NO_2 interaction with bulk KBr. The red spectrum indicates NO_2 saturation, and gray spectra represent NO_2 interaction with KBr surface while NO_2 contacted to the bulk KBr prior to NO_2 saturation. (Gas feed: 400 ppm NO_2 + Ar at 150°C, total flow rate: 100 Nml·min^{-1}, sample bed Temp.: 150°C)

References

(1) Han, J.; Wang, A.; Isapour, G.; Härelind, H.; Skoglundh, M.; Creaser, D.; Olsson, L. N2O Formation during NH3-SCR over Different Zeolite Frameworks: Effect of Framework Structure, Copper Species, and Water. *Industrial & Engineering Chemistry Research* **2021**, *60* (49), 17826-17839. DOI: 10.1021/acs.iecr.1c02732.

(2) National Institute of Standards and Technology (NIST) Chemistry WebBook, date of access: 2024-05-13. DOI: https://webbook.nist.gov/.

(3) Bordiga, S.; Regli, L.; Lamberti, C.; Zecchina, A.; Bjørgen, M.; Lillerud, K. P. FTIR Adsorption Studies of H2O and CH3OH in the Isostructural H-SSZ-13 and H-SAPO-34: Formation of H-Bonded Adducts and Protonated Clusters. *The Journal of Physical Chemistry B* **2005**, *109* (16), 7724-7732. DOI: 10.1021/jp044324b , note = PMID: 16851897.
